# Integrated CO_2_ Capture and Utilization by Combining Calcium Looping with CH_4_ Reforming Processes: A Thermodynamic and Exergetic Approach

**DOI:** 10.1021/acs.energyfuels.4c01462

**Published:** 2024-06-21

**Authors:** Theodoros Papalas, Andy N. Antzaras, Angeliki A. Lemonidou

**Affiliations:** † Department of Chemical Engineering, Aristotle University of Thessaloniki, University Campus, 54124 Thessaloniki, Greece; ‡ Department of Chemical Engineering and Biotechnology, University of Cambridge, Philippa Fawcett Drive, CB3 0AS Cambridge, U.K.; § Chemical Process & Energy Resource Institute, CPERI/CERTH, Thermi, Thessaloniki 57001, Greece

## Abstract

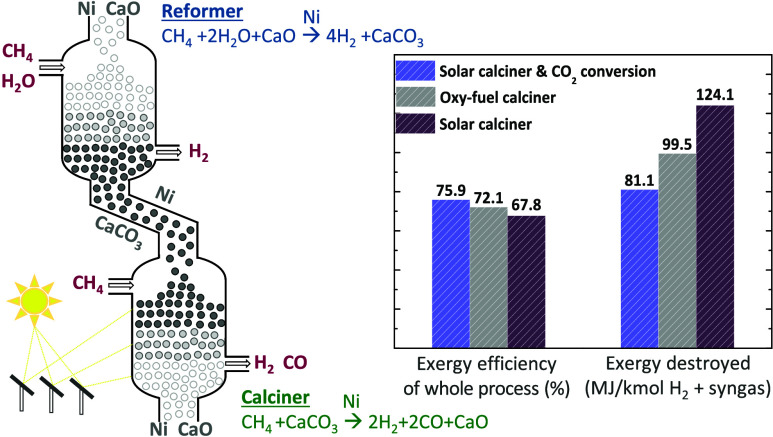

This study investigates a novel concept to coproduce high-purity H_2_ and syngas, which couples steam methane reforming with CaO carbonation to capture the generated CO_2_ and dry reforming of methane with CaCO_3_ calcination to directly utilize the captured CO_2_. The thermodynamic equilibrium of the reactive calcination stage was evaluated using Aspen Plus via a parametric analysis of various operating conditions, including the temperature, pressure, and CH_4_/CaCO_3_ molar ratio. Introducing a CH_4_ feed in the calcination stage promoted the driving force and completion of CaCO_3_ decomposition at lower temperatures (∼700 °C) compared to applying an inert flow, as a result of *in situ* CO_2_ conversion. A conceptual process design was investigated that employs a system of two moving bed reactors to produce nearly equivalent volumetric flows of pure H_2_ and a syngas stream with a H_2_/CO molar ratio close to 1. A solar reactor was examined for the reactive calcination step to cover the energy requirements of endothermic CaCO_3_ decomposition and dry reforming. The overall exergy efficiency of the process was found equal to ∼75.9%, a value ∼4.0 and ∼8.0% higher compared to sorption-enhanced reforming with oxy-fuel and solar calciner, respectively, without direct utilization of the captured CO_2_.

## 1 Introduction

Increasing CO_2_ emissions derived from the excessive use of fossil fuels have triggered an immediate need for Carbon Capture, Utilization, and Storage (CCUS) technologies. Calcium looping is a promising technology for reducing the environmental footprint of the industrial sector by separating CO_2_ from the flue gas via the exothermic carbonation of CaO (eq 1). The carbonated material can revert back to oxide form via the reverse endothermic reaction in a separate reactor.^1^ Aside from postcombustion CO_2_ capture, calcium looping can intensify thermodynamically limited reactions, such as steam methane reforming (eq 2, SMR). Conventional SMR plants without CCUS are widely deployed at industrial scale and account for ∼50% of the worldwide production of H_2_,^2^ a major building block for refineries and chemical and petrochemical industries and also an emerging energy carrier for transportation and electricity generation. However, SMR retains severe energy demand due to the harsh operation of the reformer (temperatures of 800–900 °C and pressures of 20–30 bar). Another downside is the high carbon footprint due to the large quantities of CO_2_ emitted to the atmosphere. CO_2_ is a byproduct of reforming and water gas shift (eq 3, WGS) reactions and, in addition to CO_2_ from combusting natural gas to cover the energy demand of the reformer, ends up in the flue gas of the unit.^3^ Coupling calcium looping and SMR has been established as an intensified technology, called sorption-enhanced steam methane reforming (SE-SMR). The presence of CaO enables *in situ* capture of the byproduct CO_2_ and the shift of the system toward a new thermodynamic equilibrium with high H_2_ yield and purity in a single step while operating at lower temperatures (600–650 °C). Moreover, heat released from carbonation is exploited *in situ* for reforming, avoiding external fuel combustion.^4,5^
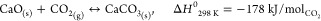
1
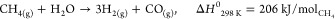
2
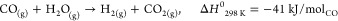
3

Despite the advantages of SE-SMR, the calcination is usually conducted under a pure CO_2_ stream in order to not dilute the CO_2_ released from CaCO_3_ decomposition, forcing the reactor to operate at harsh temperatures (≥900 °C).^6,7^ The group of *Farrauto* has proven that using a reactive gas feed instead of CO_2_ in the calciner can cause the *in situ* conversion of captured CO_2_ in the presence of a suitable catalyst and drive calcination at lower temperatures, according to *Le Chatelier’s* principle.^8,9^ An example of reactive gas is CH_4_, which can react with the captured CO_2_ via dry reforming of methane (eq 4, DRM). Apart from DRM, the reverse WGS (reverse eq 3, RWGS), CH_4_ decomposition (eq 5), and Boudouard (eq 6) reactions may occur and affect the quality of syngas.^10^
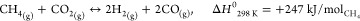
4
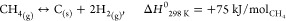
5
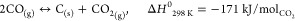
6

The potential of reducing the temperature of calcination has triggered the interest for various integrated CO_2_ capture and utilization processes,^11,12^ while calcium looping coupled with dry reforming of methane (CaL-DRM) has already been widely studied and applied for postcombustion CO_2_ capture applications.^10,13−17^ A major issue of this process is the carbon deposition in the reactive calcination stage.^16−18^ Although carbon can be gasified by CO_2_ in the subsequent carbonation stage, the release of CO with the CO_2_-stripped flue gas raises environmental concerns,^13,19^ while CO_2_ cannot completely gasify all carbon.^16^ Another problem is related to the partial oxidation of the Ni surface during exposure under CO_2_ during carbonation.^15,20^ Even though the formed NiO can be reduced from CH_4_ in the calcination stage,^13^ the inadequate number of Ni active sites in the beginning of the stage can affect the syngas quality. Finally, the CO_2_ uptake of CaO displays rapid decrease over cycles, thereby reducing the syngas production capacity.^16,17^

The challenges of CaL-DRM and the aforementioned high calcination temperatures of SE-SMR could be alleviated by coupling the carbonation and calcination steps of calcium looping with steam and dry-reforming of methane simultaneously (SMR-CaL-DRM). The proposed intensified process can take place in two reactors using CH_4_ as carbonaceous feed to produce high-purity H_2_ with *in situ* capture of CO_2_ and to directly utilize the captured CO_2_ for syngas generation. Both H_2_ and syngas comprise major building blocks for the industry and the energy sector, and they can be produced from SMR and DRM in the presence of the same Ni-based catalyst. Conducting SMR along with carbonation could enable carbon deposited in the preceding calcination-DRM stage to be more efficiently gasified from H_2_O compared to CO_2_, while the produced CO could further react via the WGS reaction (eq 3) and produce H_2_. The presence of H_2_O during carbonation could also lead to higher CO_2_ capture activity and stability of CaO over cycles compared to operating under dry conditions, thereby securing a higher syngas uptake.^7,21^ Finally, the H_2_ generated during reforming can create a strongly reducing environment and ensure that Ni is retained in metallic form for the subsequent reactive calcination stage.

The benefits of SMR-CaL-DRM dictate the necessity for further study to realize the potential of this technology. A thermodynamic analysis could indicate the optimum operating windows for the reforming and calcination stages. Despite the plethora of work on the thermodynamics of the reforming stage,^22−26^ studies dealing with the effect of operating conditions on calcination coupled with DRM are scarce.^27,28^ It is also necessary to address whether SMR-CaL-DRM can compete with SE-SMR in terms of energy efficiency, since despite the lower operating temperatures, SMR-CaL-DRM combines two highly endothermic reactions in a single stage. Conducting an exergy analysis could provide an answer to this question. Based on the second law of thermodynamics, an exergy analysis accounts for the degradation of mass and energy streams when undergoing various processes, providing a rational and meaningful assessment of the useful energy contained in a system.^24,29,30^ Zhang *et al.* reported a higher exergy efficiency for sorption-enhanced biomass gasification coupled with *in situ* conversion of CO_2_ to syngas compared to standalone sorption-enhanced gasification.^28^ Such a study would be fruitful for SMR-CaL-DRM, which has not yet been reported yet. The exergy analysis could also explore efficient ways to cover the energy demand of the highly endothermic calcination stage. Oxy-fuel calciners comprise the most widely studied solution for conventional calcium looping or the SE-SMR process. However, cofeeding O_2_ with CH_4_ in the calciner of SMR-CaL-DRM can decrease the selectivity toward DRM and the calcination driving force.^16,31^ On the other hand, solar heating comprises a sustainable approach to cover the energy demand,^32,33^ while solar reactors have been studied for thermochemical energy storage applications of calcium looping.^34,35^

In this work, Aspen Plus software is used to conduct a thermodynamic analysis of calcination coupled with DRM, along with a conceptual design and exergy analysis of the SMR-CaL-DRM process. The thermodynamic analysis aims to clarify the effect of different operating parameters, including the reactor temperature, pressure, and CH_4_ to CaCO_3_ molar ratio, on the efficiency of the reactive calcination stage. The process design is conducted by simulating both the reformer and calciner as moving bed reactors and by introducing kinetic correlations of the literature for reactions taking place. Solar heating is investigated as a means of covering the energy demand of the calcination coupled with the DRM stage, and the SMR-CaL-DRM process is evaluated from an exergetic point of view and compared to SE-SMR with either a solar or an oxy-fuel calciner. The results are expected to highlight the potential of SMR-CaL-DRM and contribute to the ongoing research on integrated CO_2_ capture and utilization processes.

## 2 Methodology

### 2.1 Thermodynamic Analysis

The effect of operating conditions on the performance of the *calcination/DRM stage* was evaluated via equilibrium calculations with the Aspen Plus V9 computational software. The thermophysical properties of all substances are defined by the *Peng–Robinson* equation of state. Equilibrium compositions are calculated using the *RGIBBS* model, by minimizing the Gibbs free energy of introduced components. A sensitivity analysis is performed for the *calcination/DRM stage*, including a range of temperatures and pressures for operating the reactor and different CH_4_/CaCO_3_ molar ratios for the inlet stream. Simulations are run by either accounting or neglecting carbon formation, while the production of compounds other than CH_4_, H_2_O, H_2_, CO, CO_2_, CaO, CaCO_3_, and C is considered nonthermodynamically favorable under the studied conditions. Table 1 summarizes all parameters investigated. For comparison, a simulation is run where calcination is conducted under inert flow (100 vol % N_2_).

**Table 1:** Range of Parameters Studied for the *Calcination/DRM Stage*

temperature (°C)	pressure (bar)	CH_4_/CaCO_3_ molar ratio (–)
500–1000	1–25	0.1–3.5

The above parameters are evaluated for their effect on CaCO_3_ and CH_4_ conversions, *in situ* CO_2_ utilization, purity, and H_2_/CO molar ratio of syngas. CaCO_3_ conversion is defined (eq 7) as the difference between inlet and outlet molar flows of CaCO_3_, divided by the inlet molar flow of CaCO_3_. CH_4_ conversion is defined similarly (eq 8) as the difference between the inlet and outlet molar flows of CH_4_, divided by the inlet molar flow of CH_4_.
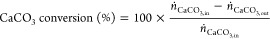
7
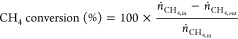
8*In situ* CO_2_ utilization efficiency is defined (eq 9) as the CO_2_ moles that are converted to syngas. This is expressed as the difference between the inlet molar flow of CaCO_3_ and outlet molar flows of CO_2_ and CaCO_3_, divided by the inlet molar flow of CaCO_3_.
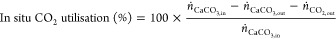
9Syngas purity is defined (eq 10) on a dry basis as the outlet molar flows of H_2_ and CO divided by the total molar flow in the outlet stream. The H_2_/CO molar ratio is also found (eq 11) by dividing the outlet molar flows of H_2_ and CO.
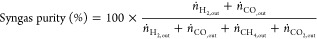
10
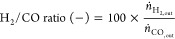
11

### 2.2 Conceptual Process Design

This section describes the methodology followed for the process design and exergy analysis. Τhe main scenario studied (case 1) comprises the integrated SMR-CaL-DRM process with solar calciner and it is compared to the SE-SMR process where calcination is performed with a solar (case 2) or an oxy-fuel heating (case 3) approach. Each process is designed for a H_2_ production capacity of ∼11,000 N m^3^/h.

#### 2.2.1 Process Flow Diagram

**Figure 1:**
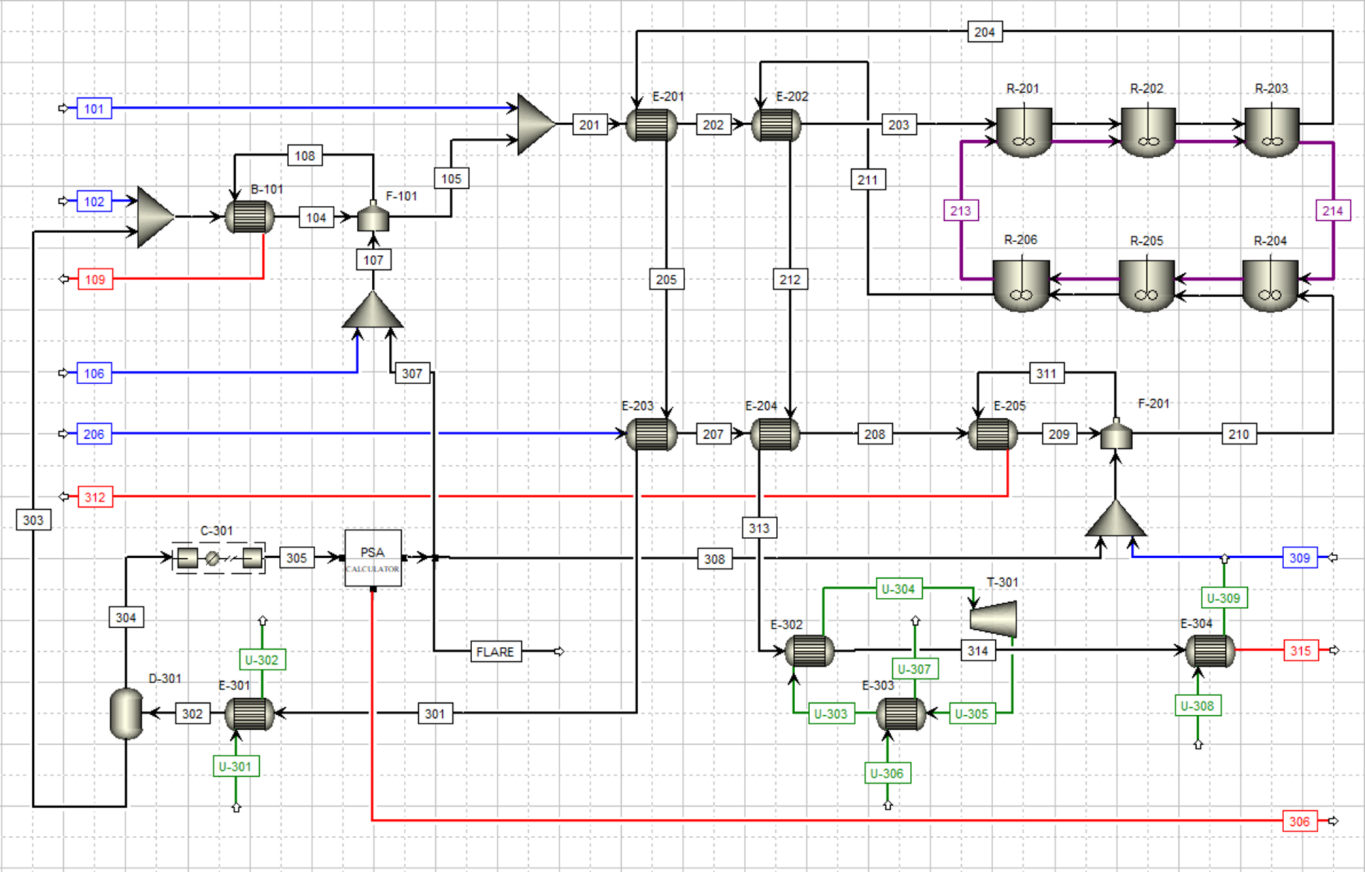
Simulation flowchart prepared for the conceptual process design (streams colored in blue refer to inlet gas streams, red to outlet gas streams, black to other gas streams, green to utility streams, and purple to solid streams circulating between the two reactors).

Figure 1 illustrates the flow diagram for the three cases, with the main difference between them being the composition of the various streams. The flow diagram consists of the following.

- Steam generation and mixing with natural gas to form the gas feed of the reformer by also recycling unreacted steam condensate from the reactor outlet.
- H_2_ production with *in situ* capture of CO_2_ in a moving bed reactor and transfer of saturated solids in a second moving bed reactor for CaCO_3_ calcination to occur.
- Heat integration of the hot gas product streams for preheating the feeds of both reformer and calciner and for generating electricity in a heat recovery steam cycle.
- Final compression and purification of H_2_ by pressure swing adsorption (PSA) and combustion of part of PSA tail gas to generate steam and preheat the feed of calciner.

In the proposed conceptual design, fresh process water (stream **102**) is mixed with recycled water (stream **303**). The required heat for steam generation in the boiler, simulated by the array of heat exchanger **B-101** and furnace **F-101**, is provided by combusting part of the PSA tail gas (stream **307**) with air (stream **106**). The generated steam is then mixed with the appropriate amount of natural gas (stream **101**) to obtain a H_2_O to CH_4_ molar ratio of 3, and the total stream is finally preheated in two sequential heat exchangers (**E-201** and **E-202**) by exploiting the heat from H_2_ (stream **204**) generated in the reformer and gas product (stream **211**) obtained from the calciner. The preheated stream (**203**) is then introduced in the reformer. Aspen Plus does not contain a built-in model to simulate the moving bed reactor. Therefore, the reformer is simulated with an array of three RCSTR models in series (**R-201**, **R-202**, and **R-203**), which represent the top, middle, and bottom of the reactor, respectively, with this approach having been previously proposed for the simulation of moving bed reactors in Aspen Plus.^36^ Stream **203** enters the reformer by being introduced to RCSTR model **R-201** together with a stream (**213**) of solid components returning from the calciner.

The spent solids of the reformer (stream **214**, exiting RCSTR model **R-203**) are then fed in the calciner, which is also simulated with an array of three RCSTR models in series (**R-204**, **R-205**, and **R-206**). The solid stream is introduced to RCSTR model **R-204** along with a gas inlet flow (stream **210**) whose composition differentiates based on the studied case (natural gas for case 1, CO_2_ for case 2, and natural gas and O_2_ for case 3), while both gas and solid components exit the reactor from RCSTR model **R-206**. The gas feedstock of the calciner (stream **206**) is initially preheated in an array of two heat exchangers (**E-203** and **E-204**) using the heat of the H_2_ and calciner gas products (streams **205** and **212**, respectively) that remains after preheating the reformer’s feed, followed by a furnace (**F-201**) that combusts part of the PSA tail gas (stream **308**).

The heat-depleted H_2_ product (stream **301**) is further cooled using cooling water in a heat exchanger (**E-301**) to condense unreacted steam, which is then separated (stream **303**) in a flash separation drum (**D-301**), in order to be recycled and reused. The gas outlet of the drum (stream **304**) is compressed to 25 bar in a two-stage compressor (**C-301**) and the compressed gas (stream **305**) is introduced to a PSA unit to remove impurities (CO_2_, unreacted CH_4_ and CO). The latter is simulated with a *calculator block* that defines the composition and flow of the pure H_2_ product (stream **306**) and PSA tail gas. Most of the tail gas of the PSA unit is used as fuel for the aforementioned preheating purposes (steam generation and preheating of the calciner inlet flow), while the remaining gas is flared to the atmosphere.

Regarding the gas outlet of the calciner (stream **211**), after preheating the gas feed of the reformer and the calciner in heat exchangers **E-202** and **E-204**, respectively, the temperature of the stream (**313**) is above 400 °C. The remaining energy is used to produce superheated steam (heat exchanger **E-302**), which is expanded and cooled down in a heat recovery cycle to generate electricity in steam turbine **T-301**. The heat-depleted stream (**314**) is then cooled using cooling water in heat exchanger **E-304**.

All simulations are conducted by considering the following assumptions.

- Natural gas is composed of 100 vol % CH_4_.
- All inlet streams are delivered at 15 °C and 1 bar, while cooling water utility is available at 20 °C and 1 bar.
- All heat exchangers are operated with counter-current flow of hot and cold streams, with a Δ*T* between the hot outlet and the cold inlet streams being 20 °C, except the preheater of the reformer (heat exchanger **E-202**).
- The reformer operates at 1 bar and under a fully adiabatic mode. The feed of the reactor is composed of H_2_O and CH_4_ with a molar ratio of 3, while the amount of CH_4_ is adequate for the CH_4_/CaO ratio to be equal to 1.
- The calciner operates at 1 bar and at 800 °C for case 1 and 900 °C for cases 2 and 3.
- The above description refers to a cocurrent configuration for the moving bed reactors. Counter-current flow is also investigated by feeding streams **214** and **215** to **R-203** and **R-206**, which results in their exit from **R-201** and **R-203**. The reactor dimensions are the same for cocurrent and counter-current flow and are specified by accounting that in the counter-current flow, the gas velocity should be lower than the minimum fluidization velocity, calculated through the correlations of Wen and Yu.^37^
- The solid material circulating between the two reactors is assumed to be a bifunctional material studied in our previous work,^16^ with a nominal composition of 60 wt % CaO, 10 wt % NiO, and 30 wt % CaZrO_3_ (in reduced state). The material is assumed to have a particle size distribution between 1.8 and 3.5 mm, while similar particle sizes have been reported before in studies with moving bed reactors.^38−40^ Deactivation of the material over consecutive cycles is considered negligible.
- Kinetic models from the literature are applied to describe the reactions taking place,^41−45^ which are presented in detail with eqs S1–S17.
- The PSA unit attains 85% H_2_ separation with a purity of 99.999 vol %.^46^
- Compressors and turbines are isentropic with an efficiency of 72%.

#### 2.2.2 Exergy Analysis

The two cases studied are further compared based on their exergetic efficiency. Standard conditions are defined as a pressure of 1 bar and temperature of 25 °C (*T*_0_). Exergy flow of a material stream *j* (Ėx_*j*_) consists of the chemical (Ėx_chem,*j*_), physical (Ėx_phys,*j*_), kinetic (Ėx_kin*,j*_), and potential (Ėx_*p,j*_) exergy flow terms (eq 12).^29^ The latter two can be neglected since neither high velocities nor large height differences are considered.^47^
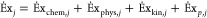
12

The chemical and physical exergy of a stream with molar flow rate *ṅ*_*j*_ can be described by eqs 13 and 14. The standard specific exergy of each component (ε_*i*_^ο^), needed for the calculation of the chemical exergy term, is provided in Table 2. For solids, chemical exergy is found like as they are in the gaseous phase.^48^ The CaZrO_3_ flow does not alter between the inlet and outlet streams of reactors and does not affect the analysis outcome.
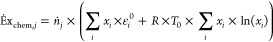
13
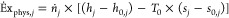
14

**Table 2:** Standard Specific Exergy for All Chemical Compounds Used (Obtained from Refs (29,49))

chemical compound (–)	standard specific exergy (MW/kmol)
CH_4_	833.9
CO	277.1
CO_2_	19.9
H_2_	236.1
H_2_O	9.5
N_2_	0.72
O_2_	3.97
CaO	127.3
CaCO_3_	16.3

Equations 12–14 enable the calculation of the exergy flows of inlet (Ėx_in,*k*_) and outlet (Ėx_out,*k*_) material streams for each equipment module *k*. Using this data, it is possible to find the exergy flow destroyed (Ėx_des,*k*_) in module *k* with the equations of Table 3.

**Table 3:** Exergy Balances of Individual Modules

module	exergy balance
heat exchangers	Ėx_des,*k*_ = (Ėx_in,*k*_ – Ėx_out,*k*_)_hot fluid_ + (Ėx_in,*k*_ – Ėx_out,*k*_)_cold fluid_
furnaces	Ėx_des,*k*_ = (Ėx_in,*k*_ – Ėx_out,*k*_)_fuel_ + (Ėx_in,*k*_ – Ėx_out,*k*_)_heated fluid_
compressors	Ėx_des,*k*_ = Ėx_in,*k*_ + *Ẇ*_comp_ – Ėx_out,*k*_
turbines	Ėx_des,*k*_ = Ėx_in,*k*_ – *Ẇ*_turb_ – Ėx_out,k_
steam reformer and oxy-fuel calciner	Ėx_des,*k*_ = (Ėx_in,*k*_ – Ėx_out,*k*_)_gas_ + (Ėx_in,*k*_ – Ėx_out,*k*_)_solid_
solar calciner	Ėx_des,*k*_ = Ėx_*Q*,calc_ + (Ėx_in,*k*_ – Ėx_out,*k*_)_gas_ + (Ėx_in,*k*_ – Ėx_out,*k*_)_solid_
mixing streams and PSA unit	Ėx_des,*k*_ = Ėx_in,*k*_ – Ėx_out,*k*_

Work and heat streams also affect the exergy balance of each module. Electricity needed and produced in the compressor and the turbine, respectively, can be considered equivalent to work (*Ẇ*_comp_ and *Ẇ*_turb_) and exergy flow. However, heat flow (*Q̇*_s_) provided by a heating source with temperature *T* is associated with exergy destruction (Ėx_*Q*,calc_), since not all heat can be used for work (eq 15).^24,30^
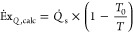
15Heat is required for only the solar calciners of cases 1 and 2. Calcium looping driven by concentrated solar power has been widely studied in the literature, in which a heliostat field directs solar radiation toward a tower receiver, which can be the calciner itself.^48,50,51^ In general, solar irradiation *Q̇*_s_ depends on the surface area *A* of the heliostat field and on direct normal irradiance DNI, with the latter varying between geographic regions and time zones. The heat received by the tower *Q̇*_tower_ is a function of *Q̇*_s_ and the efficiency of the heliostat field (*n*_helio_). Except for heliostat losses, heat lost due to radiation or reflection from the outer surface of the receiver and during the transfer of heat from the outer surface to the material within the reactor can also affect the heat exploited in the *calcination/DRM stage* (*Q̇*_calc_). These losses can be accounted with the efficiency of the tower receiver *n*_rec_.^51^Equation 16 shows the relation between all aforementioned terms.
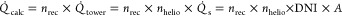
16The *Q̇*_calc_ term can be retrieved from the Aspen Plus simulations, while *n*_rec_ and *n*_helio_ are assumed to be equal to 80 and 85%, respectively, which are average values from the literature.^48,50−53^ This data allows the calculation of *Q̇*_s_ and Ėx_*Q*,calc_, by defining *T* to be equal to 5300 °C, the temperature of the outer surface of the sun.^47^ The heliostat field area can also be estimated by assuming an average value of 700 W/m^2^ for DNI.^52^ It should be stressed that this study focuses on the conceptual design of the process, while a more detailed design of the heliostat and the reactor would be needed for realization and accurate estimation of *n*_rec_ and *n*_helio_ efficiencies, rendered out of the scope of this work.

After finding the Ėx_des,*k*_ for each module *k*, the exergy efficiency *n*_ex_ can be found with eq 17. The Ėx_in,tot_ and Ėx_out,total_ terms refer to the total input and output exergies from material streams and they can be found from eqs 18 and 19. Streams contributing to the total input and output exergy flows are marked with blue and red color in Figure 1.
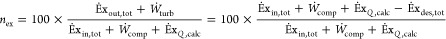
17
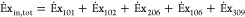
18
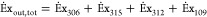
19

## 3 Results and Discussion

### 3.1 Thermodynamic Analysis

Thermodynamic calculations are conducted using the *RGIBBS* model of Aspen Plus in order to find the optimum operating conditions for the *calcination/DRM stage*. The section below describes the alteration of the different performance indicators listed in eqs 7–11 as a function of the temperature, pressure, and CH_4_/CaCO_3_ molar ratio when not accounting for carbon formation. Separate simulations are also conducted to consider carbon as the possible product. The main outcome of all simulations comprises the outlet molar flow composition of the *RGIBBS* model, which then allows for the calculation of the performance indicators. The outlet molar flow composition for all simulations run, along with the results of the thermodynamic analysis when accounting for carbon formation, are presented in Figures S1 and S4.

**Figure 2:**
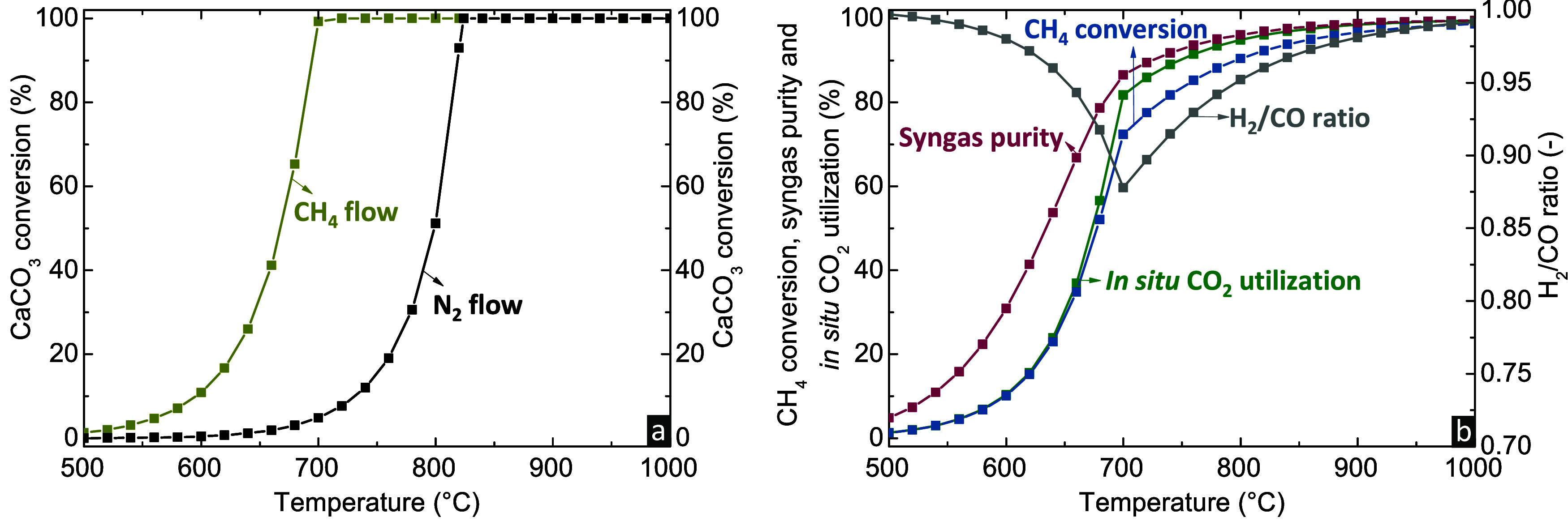
Effect of temperature on (a) CaCO_3_ conversion when applying pure CH_4_ or pure N_2_ gas feed and (b) CH_4_ conversion, *in situ* CO_2_ utilization, purity, and H_2_/CO molar ratio of the produced syngas when applying pure CH_4_ gas feed (*P* = 1 bar, CH_4_/CaCO_3_ = 1, no carbon formation).

Figure 2a compares the CaCO_3_ conversion as a function of temperature, when exposing CaCO_3_ under a N_2_ or CH_4_ gas flow, which reveals the clear advantage of the intensified *calcination/DRM stage*. Applying the reactive CH_4_ flow leads to the *in situ* consumption of CO_2_ via DRM (eq 4) or RWGS (reverse eq 3) reactions. The decrease of CO_2_ partial pressure due to the *in situ* conversion and volumetric increase caused by the DRM reaction (eq 4) enhance the driving force of CaCO_3_ calcination, causing the system to shift toward a different thermodynamic equilibrium compared to the case where calcination is performed under an inert gas (N_2_) flow. Higher temperatures promote the calcination extent due to the endothermic nature of the reaction. Ultimately, full CaCO_3_ conversion is attained at 700 °C, a much lower temperature compared to applying the N_2_ gas flow. It is acknowledged that a N_2_ gas flow is not a realistic operation for conventional calcination compared to that using pure CO_2_. For the purpose of this study, N_2_ is used as an inert gas flow to clearly demonstrate the enhanced calcination driving force when applying a reactive CH_4_ gas flow. It should also be mentioned that using a pure CO_2_ feed would not permit conversion of CaCO_3_ thermodynamically until the temperature would exceed 900 °C,^43^ a difference of more than 200 °C compared to full CaCO_3_ conversion under CH_4_ flow.

Figure 2b demonstrates the alteration of the remaining performance indicators (CH_4_ conversion, *in situ* CO_2_ utilization, syngas purity, and H_2_/CO molar ratio) as a function of temperature when applying pure CH_4_ flow. For temperatures up to 700 °C, where CaCO_3_ is incomplete and the molar ratio of CH_4_ to CO_2_ released from calcination is substoichiometric, CH_4_ conversion and CO_2_ utilization increase exponentially with temperature, with both being mainly dictated by the CO_2_ released from CaCO_3_ calcination. At temperatures above 700 °C, where full calcination is attained, CH_4_ conversion and CO_2_ utilization increase rates decline and are solely affected by the extent of the DRM and RWGS reactions at the respective temperature. It is noted that at the threshold of 700 °C, the CH_4_ conversion and *in situ* CO_2_ utilization exceed 70 and 80%, respectively.

The purity of the generated syngas follows a trend similar to that of the other performance indicators. In the temperature range of 500–700 °C, it sharply increases from ∼5 to ∼86%, with the remaining percentage of the gas phase mainly consisting of unreacted CH_4_. For temperatures above 700 °C, where CaCO_3_ conversion is equal to 100%, the system is mainly affected by the equilibrium of DRM and RWGS. An increase in the temperature enhances the CH_4_ and CO_2_ conversions and thus the purity of the produced syngas. Finally, the H_2_/CO molar ratio presents an inverse volcano profile versus temperature, with the peak occurring at the point of complete CaCO_3_ calcination. As the temperature increases up to 700 °C, the ratio of CH_4_ to CO_2_ released from CaCO_3_ calcination remains above the stoichiometric value for DRM reaction. CO_2_ is the limiting reactant in this range and part of it reacted with the produced H_2_ via the RWGS reaction toward generating CO and H_2_O. The mildly endothermic RWGS seems to be favored over the strongly endothermic DRM until reaching 700 °C. Above the breakpoint of 700 °C, an increase of temperature led to the expected gradual increase of the H_2_/CO molar ratio close to unity, as the DRM reaction proceeded to a higher extent compared to RWGS.

Overall, all reactions are interconnected with each other, and the temperature of the *calcination/DRM stage* can highly affect their extent and selectivity. Due to the important role of temperature in the efficiency of the *calcination/DRM stage*, the effect of other parameters is investigated at three different temperatures, which include the breakpoint temperature for complete calcination (700 °C), a low temperature where CaCO_3_ decomposition is limited (650 °C), and an elevated temperature (800 °C), where both CaCO_3_ and CH_4_ conversions are high.

**Figure 3:**
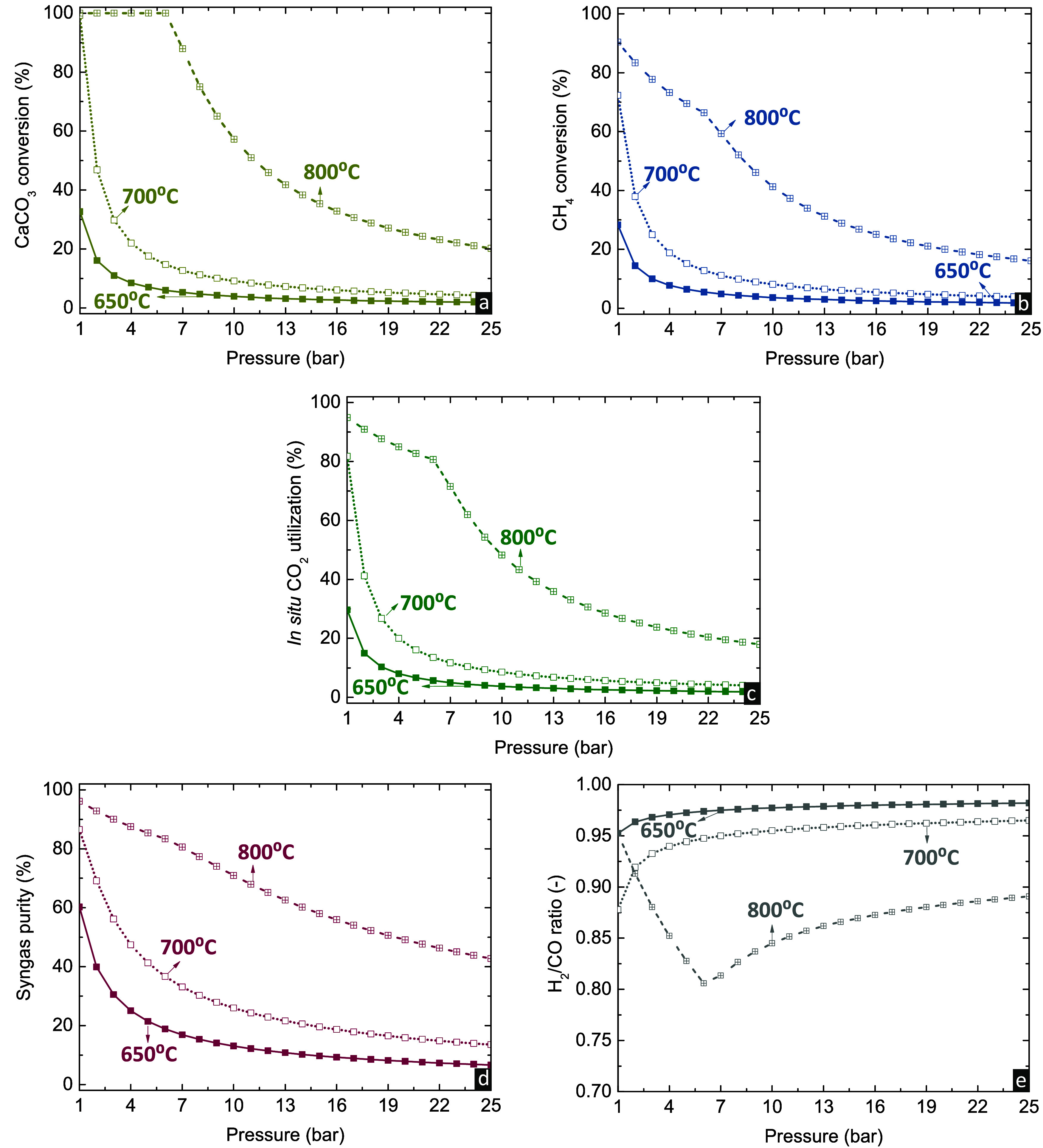
Effect of pressure on (a) CaCO_3_ conversion, (b) CH_4_ conversion, (c) *in situ* CO_2_ utilization, (d) purity, and (e) H_2_/CO molar ratio of the produced syngas in the *calcination/DRM stage* (CH_4_/CaCO_3_ = 1, no carbon formation).

Figure 3 illustrates the effect of pressure on different performance indicators of the *calcination/DRM stage*. CaCO_3_ conversion presents a gradual decrease with increasing pressure at 650 and 700 °C due to the increase of partial pressure of CO_2_, which is the only gaseous product of the reaction. A constant full conversion precedes the aforementioned decrease for pressures up to ∼6 bar at 800 °C, which is the reason for the other performance indicators presenting two different regimes as a function of the pressure at this temperature. The first regime is related to full CaCO_3_ conversion and stoichiometric molar ratio of CH_4_ to CO_2_ released from calcination for pressures up to ∼6 bar, while for higher pressures, the decreasing CaCO_3_ conversion infers a decreasing CO_2_ to CH_4_ molar ratio as well, which affects the result.

Pressure increase has a negative effect on CH_4_ conversion, given the volumetric increase inferred by the DRM reaction. At 800 °C, where complete calcination is attained, the negative effect of pressure on CH_4_ conversion is milder, since the presence of stoichiometric CO_2_ released from calcination promotes the extent of the reaction. After passing the threshold of ∼6 bar, the CH_4_ conversion is affected by both the negative effect of pressure on the DRM reaction and the decreasing CO_2_ content, thereby leading to a more pronounced effect of pressure, similar to 650 and 700 °C. The *in situ* CO_2_ utilization and syngas purity follow the same trend as CH_4_ conversion with increasing pressure, with unreacted CH_4_ being the main impurity in the syngas.

Lastly, the pressure increase has a different effect on the H_2_/CO molar ratio for the various temperatures studied, depending on the extent of CaCO_3_ conversion. At 650 and 700 °C, where CaCO_3_ calcination and therefore release of CO_2_ are largely suppressed with increasing pressure, the H_2_/CO ratio is relatively stable. The lower H_2_/CO molar ratio at 700 °C compared to 650 °C could be attributed to the extent of RWGS, which, in contrast to DRM is only affected by temperature and not pressure changes, as it is equimolar on reactants and products. However, as both DRM and calcination reactions are negatively affected by the pressure increase, the lower amounts of H_2_ and CO_2_ shift the RWGS equilibrium toward the reactants side, leading to an increasing H_2_/CO molar ratio as a function of pressure. At 800 °C and pressures up to ∼6 bar (complete CaCO_3_ calcination), the H_2_/CO molar ratio decreases due to the high partial pressure of CO_2_, which shifts the RWGS toward the side of the products, while DRM is inhibited by the pressure increase. When pressures reach above ∼6 bar at 800 °C, the H_2_/CO molar ratio increases due to the indirect influence of the DRM and calcination extents on RWGS.

**Figure 4:**
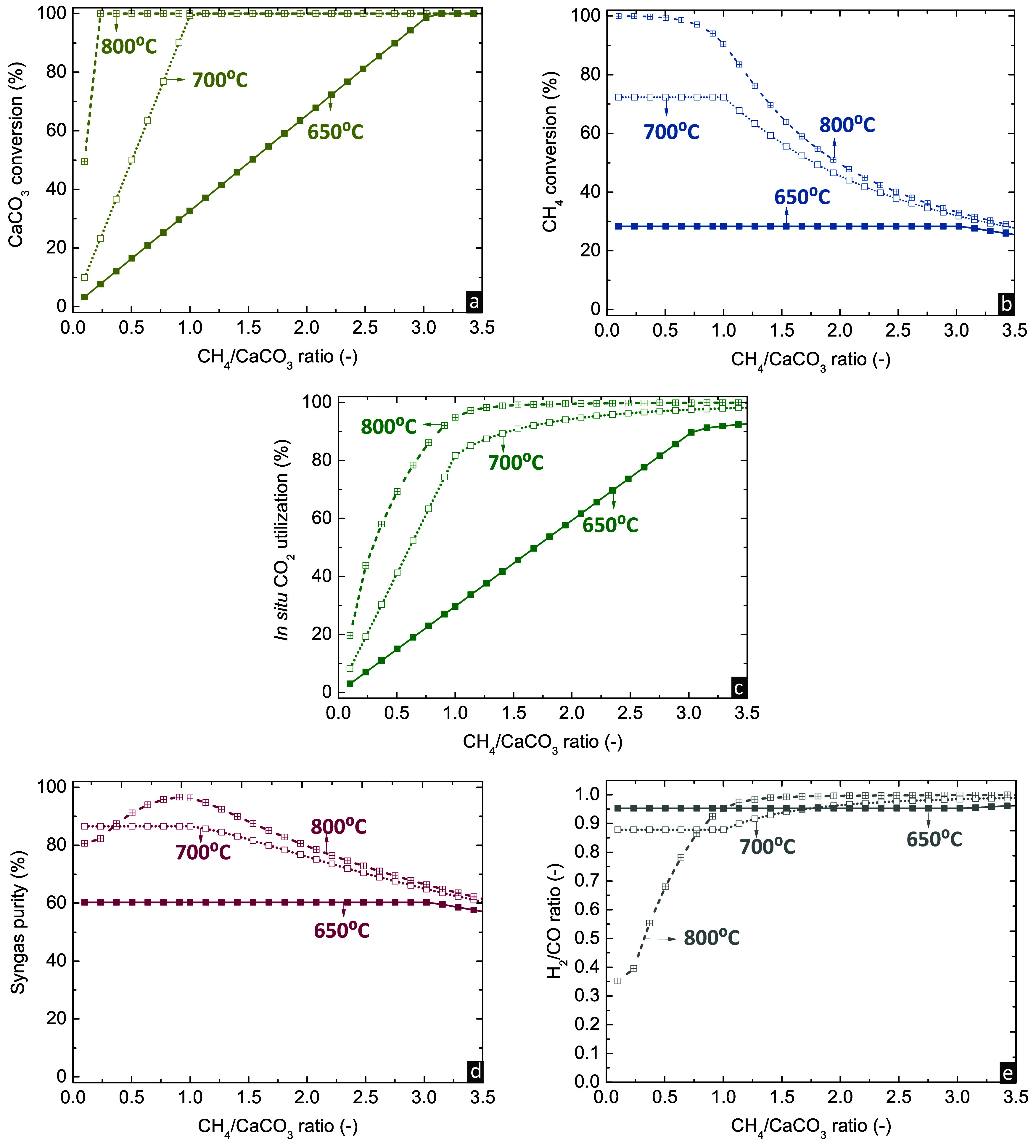
Effect of CH_4_/CaCO_3_ molar ratio on (a) CaCO_3_ conversion, (b) CH_4_ conversion, (c) *in situ* CO_2_ utilization, (d) purity, and (e) H_2_/CO molar ratio of the produced syngas in the *calcination/DRM stage* (*P* = 1 bar, no carbon formation).

As CH_4_ is the only reactant in the gas feed of the *calcination/DRM stage*, variation of the CH_4_/CaCO_3_ molar ratio can considerably affect the efficiency of the stage (Figure 4). The increase in the CH_4_ feed enhances the *in situ* CO_2_ consumption and CaCO_3_ conversion, with more CO_2_ needing to be released to reach the equilibrium partial pressure. Higher temperatures require lower CH_4_/CaCO_3_ molar ratios for full CaCO_3_ conversion, since calcination can proceed under a higher CO_2_ partial pressure in the gas phase. On the other hand, CH_4_ conversion is initially stable as a function of the CH_4_/CaCO_3_ molar ratio when operating at either 650 or 700 °C, indicating that the increase of the CH_4_ flow causes the release of an adequate amount of CO_2_ from CaCO_3_ decomposition to retain stable CH_4_ conversion. Upon reaching full CaCO_3_ conversion, the molar ratio of CH_4_ to CO_2_ released from calcination is above the stoichiometric one, leading to a gradual decrease of CH_4_ conversion from this point onward. At 800 °C, CH_4_ conversion remains at values higher than 94% for substoichiometric molar ratios (CH_4_/CaCO_3_ < 1) due to the high reaction temperature and availability of CO_2_, which both promote the DRM.

The *in situ* CO_2_ utilization displays an increasing trend as a function of CH_4_/CaCO_3_ molar ratio, similar to CaCO_3_ conversion, at 650 or 700 °C. The increase of CO_2_ utilization and the stable CH_4_ conversion until reaching full CaCO_3_ conversion prove that CO_2_ is the limiting agent that controls the equilibrium, resulting in the production of syngas with stable purity and H_2_/CO molar ratio close to unity. After reaching full CaCO_3_ conversion, the CO_2_ utilization continues to slightly increase, since the excess CH_4_ enhances the extent and selectivity of DRM compared to RWGS, as also indicated by the increase of the H_2_/CO molar ratio. However, excess CH_4_ decreases the purity of the syngas. At 800 °C and CH_4_/CaCO_3_ molar ratios lower than unity, CO_2_ utilization and syngas purity are dictated by the enhancement of DRM and RWGS with higher CH_4_ contents, resulting in a different increasing trend compared to 650 and 700 °C, where the CaCO_3_ conversion extent also affects the CO_2_ utilization. The H_2_/CO molar ratio of syngas is lower at these conditions due to the presence of unconverted CO_2_.

Overall, the results of the thermodynamic analysis provide the operating conditions for successfully integrating CaCO_3_ calcination and DRM reactions in a single step. Full CaCO_3_ conversion requires a minimum temperature of ∼700 °C for CH_4_/CaCO_3_ = 1 while attaining an *in situ* CO_2_ utilization of ∼80% toward the production of syngas with H_2_/CO molar ratio close to unity. Pressure increase results in lower conversions as neither CaCO_3_ calcination nor DRM are favored, indicating that pressures close to atmospheric are the optimum operating conditions for this integrated process. The CH_4_/CaCO_3_ molar ratio should be appropriately adjusted for the molar ratio of inlet CH_4_ to released CO_2_ to be close to unity. The aforementioned results are obtained without accounting carbon as a possible product of the thermodynamic analysis. As mentioned before, separate simulations are run where carbon formation is considered, with results being presented in Figures S1 and S2. Carbon formation is promoted at low temperatures (660–680 °C) from a thermodynamics point of view, which highlights the necessity for appropriate catalysts that can kinetically suppress the extent of carbon formation in a potential demonstration of the process. Given the potential to suppress carbon formation in a real-life application of the SMR-CaL-DRM process by operating the *calcination/DRM stage* at high temperatures and by using appropriate catalysts, carbon generation is not further studied in this work and the subsequent process design.

### 3.2 Process Design

Aspen Plus is then applied to propose a conceptual design for the SMR-CaL-DRM process. A comprehensive analysis is provided for the results from the design of case 1 of interest, where the reforming and *calcination/DRM* stages are conducted in a system of two moving bed reactors. The integrated process is then compared to the SE-SMR in terms of exergy efficiency. The conditions of each stream of the flow diagram of SMR-CaL-DRM (case 1) and SE-SMR with a solar (case 2) or an oxy-fuel calciner (case 3) are presented in Tables S4–S6.

#### 3.2.1 Performance of SMR-CaL-DRM in a System of Two Moving Bed Reactors

Setting ∼11,000 Nm^3^/h of H_2_ as a desirable production target and a H_2_O/CH_4_ molar flow ratio of 3 for operating the reformer can define the inlet volumetric flow of the reactor. The diameter is chosen to be equal to ∼1.46 m to avoid fluidization of the solid particles when designing the reactor as a moving bed with counter-current flow of gas and solids. Based on the applied kinetic correlations, the reactor has a total volume of ∼7.5 m^3^ (height of ∼4.4 m) to attain at least 90% CH_4_ conversion.

**Figure 5:**
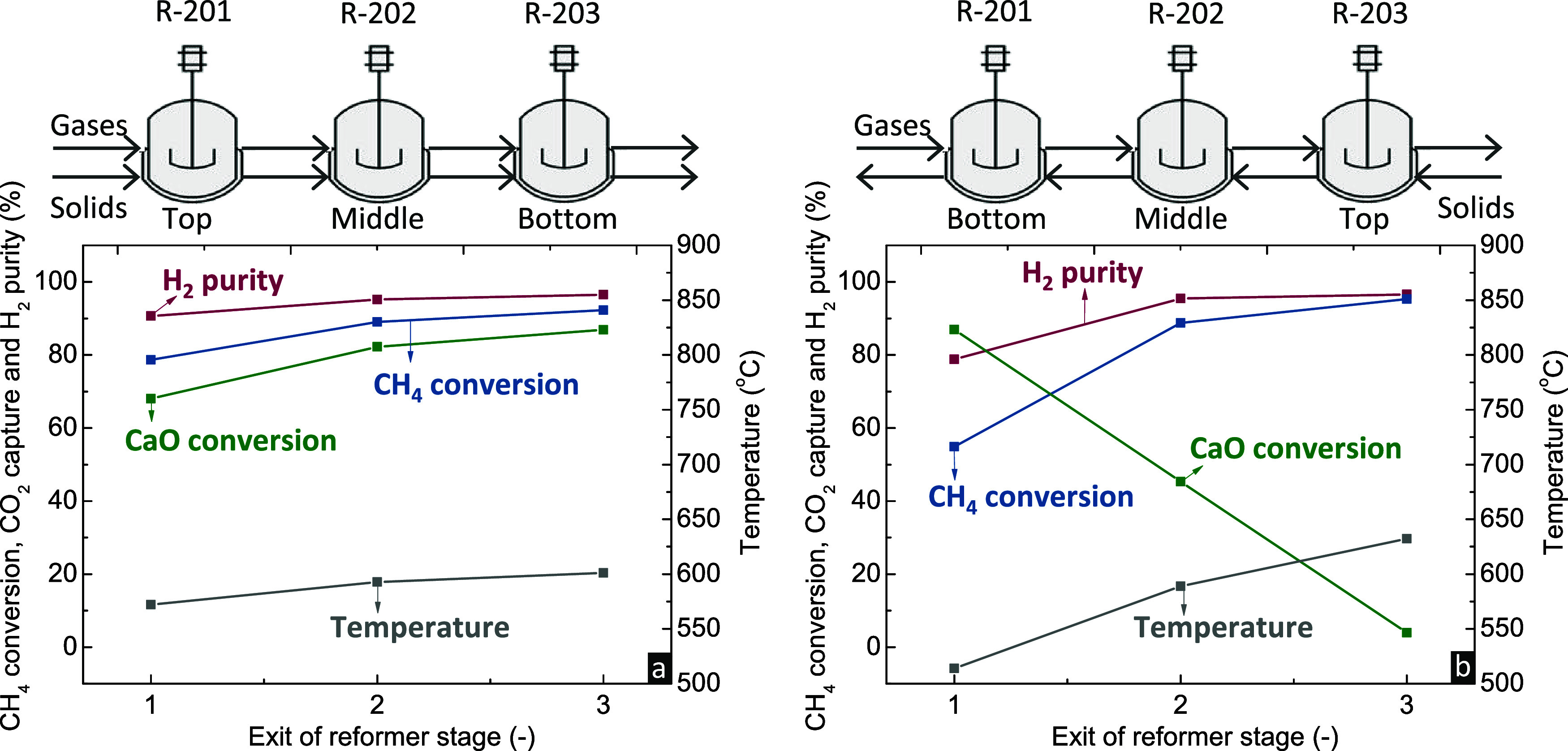
CH_4_ conversion, H_2_ purity, CO_2_ capture, and temperature profiles in the axial direction of the reformer moving bed reactor with (a) cocurrent and (b) counter-current flow of gas and solid compounds (*P* = 1 bar, H_2_O/CH_4_ = 3, CH_4_/CaO = 1). Schematics on top of the figures display the part of the reactor that each unit represents.

The reformer operates adiabatically, with the inlet gas feedstock preheated at 630 °C and the solid components circulating back from the calciner at 800 °C. Figure 5 presents the temperature, CH_4_ conversion, H_2_ purity, and CaO conversion profiles in the axial direction of the reformer with a cocurrent flow of gas and solid components. The aforementioned performance indicators are calculated based on eqs S18 and S20. In the cocurrent flow (Figure 5a), where both gases and solids enter from the top of the reactor and move downward, CH_4_ conversion and CO_2_ capture occur simultaneously, since the inlet CH_4_ comes in contact with CaO from the calciner. CO_2_ capture boosts the driving force of WGS and SMR, thereby leading to ∼80% CH_4_ conversion and ∼90% purity of the generated H_2_ at the exit of the first RCSTR model. Conversions of CH_4_ and CaO continue as the gas and solid compounds transcend the reactor, while at the bottom, ∼90% CH_4_ has been consumed to produce H_2_ with ∼95% purity. Throughout the reactor length, temperature ranges between 570 °C at the upper part of the reactor, where the endothermic reforming occurs to a higher extent, and 600 °C at the bottom.

When simulating the reformer as a moving bed with counter-current flow (Figure 5b), solid compounds perform a downward flow within the reactor and meet the gaseous components that move upward. Even though the final CH_4_ conversion, H_2_ purity, and CaO conversion are similar to the ones attained with cocurrent flow, the axial temperature profile inside the reactor is broader. Gaseous CH_4_ enters from the bottom and comes in contact with descending solids, which, however, under steady-state conditions, are already in a partially carbonated form. Therefore, SMR occurs without its heat demand being completely covered from CaO carbonation, leading to the decrease of temperature at ∼510 °C. Furthermore, SMR is not intensified to a high extent, leading to lower CH_4_ conversion and H_2_ purity compared to the cocurrent flow. As the gaseous compounds reach higher heights, they come in contact with more unreacted CaO. Since the extent of CaO carbonation is higher than SMR at the upper parts of the reactor, this leads to a temperature increase up to ∼630 °C.

The cocurrent flow of gas and solid components leads to a more uniform temperature profile, with 30 °C difference between the top and the bottom of the reactor and both the gaseous and solid compounds being retrieved at ∼600 °C. The cocurrent flow also attains an optimum coupling of SMR and CaO carbonation. On the other hand, the counter-current flow moving bed reactor results in a temperature difference of ∼120 °C between the top and the bottom of the reactor, the exit of the gaseous and solid compounds at different temperatures (∼510 and ∼630 °C, respectively), and the nonefficient coupling of SMR and CaO carbonation. The low temperature of solids and the higher difference compared to the operating temperature of the *calcination/DRM stage* (800 °C) would result in an undesirable higher energy demand for the latter. Due the aforementioned statements, the moving bed reactor with cocurrent flow of gaseous and solid compounds is deemed a more appropriate configuration for the reformer reactor. The reformate gas undergoes cooling, while after purification, a stream with ∼11,010 N m^3^/h flow and 99.999 vol % H_2_ composition is retrieved, reaching the production target.

Solid materials that exit the reformer comprise Ni, CaCO_3_, CaZrO_3_, and unreacted CaO and enter the calciner with a stream of pure CH_4_. The reactor is designed with a diameter of ∼1.65 m and a length of ∼4.18 m and operates isothermally at 800 °C and a cocurrent flow of gas and solid components. Counter-current flow is not simulated for the calciner, since the introduction of CH_4_ from the bottom would result in its contact with already partially calcined material, and the substoichiometric molar ratio of CO_2_ released from calcination to CH_4_ would promote carbon formation. The operating temperature is chosen based on the results of the thermodynamic analysis to allow high CH_4_ and CO_2_ conversion toward syngas generation (Figure 2). Cocurrent flow of solids and gas components enables to attain 97% CH_4_ conversion toward syngas production with ∼12,130 N m^3^/h volumetric flow and H_2_/CO molar ratio close to unity. The CaCO_3_ reaches full decomposition at the bottom of the reactor, attaining an *in situ* CO_2_ utilization of 98%. To perform the two endothermic reactions, a total of ∼16.3 MW or ∼116 MJ per kmol of syngas produced is required for isothermal operation at 800 °C, which would require a heliostat field of approximately 0.04 km^2^. The area of the heliostat field could be specified accurately depending on the geographic region of the unit and the DNI value.

#### 3.2.2 Exergy Analysis Results

The main inlet and outlet flows considered in the exergetic analysis for the material streams, work of compressors and turbines, and energy required in the calciner are presented in Table 4 and compared between the three cases. The SMR-CaL-DRM unit (case 1) is characterized by a higher inlet exergy flow for the material streams (Ėx_in,tot_), since more CH_4_ is required in the integrated reactive calciner compared to the amount of CH_4_ needed for the oxy-fuel calciner of case 3, while no CH_4_ is required for the solar calciner of case 2. Moreover, the aforementioned requirement for 16.30 MW of energy for operating the endothermic *calcination/DRM* stage (*Q̇*_calc_) corresponds to the required solar irradiation (*Q̇*_s_) of 23.97 MW and an inlet exergy flow of 22.69 MW (Ėx_*Q*,calc_) for case 1. The inlet exergy flow for solar radiation is lower for case 2 since only the calcination reaction occurs in the reactor, while no exergy flow of heat is required for the autothermal oxy-fuel calciner (case 3). Despite the much higher total inlet exergy flow for case 1, the retrieval of two high-value products (high-purity H_2_ and syngas) results in much higher outlet exergy flow from material streams (Ėx_out,tot_) compared to both case 2 and case 3 and a slightly higher (by 8 and 4%) overall exergy efficiency of the whole process (75.92% instead of 67.81 and 72.10%). Furthermore, the total exergy destroyed in case 1 is equal to 81.06 MJ/kmol of high-value products, which is ∼22.7 and ∼18.5% lower compared to the respective exergy destruction in case 2 and case 3.

**Table 4:** Main Exergy Results for the SMR-CaL-DRM and SE-SMR Processes

exergy term	SMR-CaL-DRM with solar calciner (case 1)	SE-SMR with solar calciner (case 2)	SE-SMR with oxy-fuel calciner (case 3)
*Ẇ*_turb_ (MW)	0.18	0.19	0.16
*Ẇ*_comp_ (MW)	2.18	2.18	2.18
*Ė*_*Q*,calc_ (MW)	22.69	11.20	0
Ėx_in,tot_ (MW)	64.57	35.71	43.21
Ėx_out,tot_ (MW)	67.70	33.10	32.56
*n*_exergy_ (%)	75.92	67.81	72.10
Ėx_des,tot_	81.06 (MJ/kmol H_2_ + syngas)	124.10 (MJ/kmol H_2_)	99.47 (MJ/kmol H_2_)

**Figure 6:**
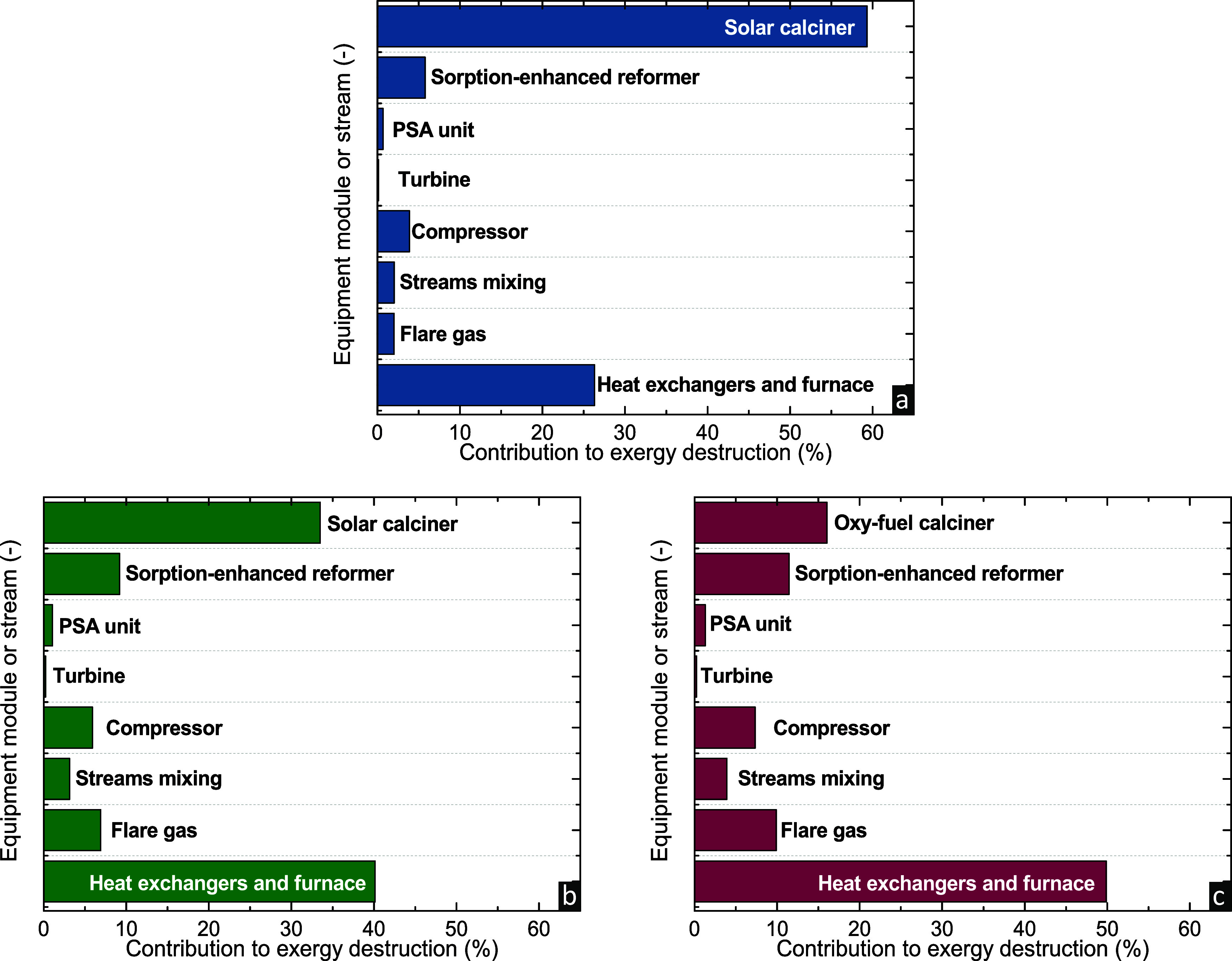
Distribution of exergy destroyed in (a) SMR-CaL-DRM process with a solar calciner and the SE-SMR process with (b) solar or (c) oxy-fuel calciner.

Figure 6 breaks down the contribution of different modules in the exergy destroyed in each case. The dominant section contributing to the exergy destruction for case 1 is the solar calciner (≥55%) as a result of heat needed to drive the two endothermic reactions. This is followed by the heat exchangers (≥25%) and more specifically the boiler needed to generate the steam for the reformer (simulated by heat exchanger **B-101** coupled with furnace **F-101**). The remaining exergy destruction (∼20%) is attributed to the reformer, the operation of the compressor and turbine units, the mixing of CH_4_ and H_2_O streams for the reformer, and the nonexploited content of the flare gas. In case 2, the solar calciner has a lower contribution to the total exergy destroyed since there is no significant chemical exergy change between the inlet and outlet streams, while the energy needed is much lower compared to the calciner of case 1. In case 3, heat exchangers are considered the main source of exergy destruction (∼50%), followed by the calciner whose exergy destruction is a result of the conversion of a stream with high chemical exergy (mixture of CH_4_ and O_2_) to a stream with low chemical exergy (mixture of CO_2_ and H_2_O). The lower contribution of the oxy-fuel calciner to the exergy destroyed compared to case 1 and case 2 signifies that future research could be focused on improving the efficiency of solar calcination and on a more detailed design of such reactors. Nonetheless, despite solar calciners being currently less promising compared to oxy-fuel calciners, the slightly higher exergy efficiency of SMR-CaL-DRM (case 1) indicates that even though two highly endothermic reactions are coupled in a single step, reactive calcination with solar heating can present a more attractive option.

## 4 Conclusions

Coupling steam methane reforming with calcium looping can lead to *in situ* removal of generated CO_2_ and elevated CH_4_ conversion toward high-purity H_2_ production in a single step. Commercializing the sorption-enhanced reforming technology relies on finding efficient methods to moderate the elevated temperatures of calcination. This work investigated an intensified process that integrates a reactive calciner in sorption-enhanced reforming to *in situ* utilize the captured CO_2_, by coupling calcination with dry reforming of methane. With both reformer and calciner being fed with CH_4_, this process coproduces high-purity H_2_ and syngas. The concept was evaluated from a thermodynamics point of view, focusing on the operation of the calcination stage, followed by a preliminary design of the integrated process while employing a system of two moving bed reactors. Solar heating was evaluated as a means of covering the energy demand of the calciner by conducting an exergy analysis to compare the proposed integrated process with a sorption-enhanced steam methane reforming process with either a solar or an oxy-fuel calciner. The main outcomes of this work are presented below.

- The complete decomposition of CaCO_3_, along with ∼80% *in situ* utilization of CO_2_ toward syngas generation were feasible thermodynamically at 700 °C, a milder temperature than conventional calcination (800–900 °C). The lower operating temperature proved that the reactive gas feed enhances the driving force of calcination.
- Cocurrent flow of gases and solids in a reformer with a moving bed configuration enabled for the adiabatic production of ∼11,000 N m^3^/h H_2_ at 600 °C, while an isothermal operation of the calciner at 800 °C resulted in the generation of similar amount of syngas (∼12,130 N m^3^/h) with H_2_/CO molar ratio close to unity. Counter-current flow was related to a more expanded profile of temperature in the axial direction of the reformer.
- Solar calcination was linked with higher exergy destruction compared to an oxy-fuel calciner. However, the *in situ* conversion of CO_2_ toward syngas allowed for the integrated proposed process to display an efficiency of ∼75.9%, a value ∼8 and ∼4% higher compared to the benchmark process with the solar and oxy-fuel calciner.

The results of this work highlighted the potential of the proposed process and intensified the interest for its experimental demonstration in the future.

## Supporting Information Available

Kinetic rate equations used for process design simulations, results of thermodynamic analysis when accounting carbon formation, molar flow composition of the outlet of the *calcination/DRM stage* as a function of the temperature, pressure, and CH_4_/CaCO_3_ (PDF)

## Nomenclature

*A*: area of heliostat field
DNI: direct normal irradiance
Ėx_des,*k*_: exergy flow destroyed in module *k*
Ėx_des,tot_: total exergy flow destroyed from modules and flare gas
Ėx_*j*_: exergy flow of stream *j*
Ėx_chem,*j*_: chemical exergy flow of stream *j*
Ėx_in,*k*_: total exergy flow of inlet material streams of module *k*
Ėx_in,tot_: total input exergy flow in the case studied
Ėx_kin*,j*_: kinetic exergy flow of stream *j*
Ėx_out,*k*_: total exergy flow of outlet streams of module *k*
Ėx_out,tot_: total output exergy flow in the case studied
Ėx_p,*j*_: potential exergy flow of stream *j*
Ėx_*Q*,calc_: exergy flow of heat required in the calciner
Ėx_phys,*j*_: physical exergy flow of stream *j*
*h*_*j*_: enthalpy of stream *j*
*h*_0*,j*_: enthalpy of stream *j* at standard conditions
*n*_ex_: exergy efficiency of the studied case
*n*_helio_: efficiency of heliostat field
*ṅ*_*j*_: molar flow of stream *j*
*ṅ*_*i*,in_: inlet molar flow of component *i*
*ṅ*_*i*,out_: outlet molar flow of component *i*
*n*_rec_: efficiency of heat transfer in the calciner
*Q̇*_calc_: heat flow required for reactions occurring inside the calciner
*Q̇*_s_: heat flow provided by a heating source
*Q̇*_tower_: heat flow reaching the outer surface of the calciner receiver
*P*: pressure
*R*: ideal gas constant
*s*_*j*_: entropy of stream *j*
*s*_0*,j*_: entropy of stream *j* at standard conditions
*T*: temperature
*T*_0_: temperature at standard conditions
*Ẇ*_comp_: work of compressor
*Ẇ*_turb_: work of turbine
*x*_*i*_: molar fraction of component *i*
ε_*i*_^0^: standard specific exergy of component *i*

## Abbreviations

CCUS: carbon capture utilization and storage
CaL-DRM: calcium looping coupled with dry reforming of methane
DRM: dry reforming of methane
PSA: pressure swing adsorption
RWGS: reverse water gas shift
SE-SMR: sorption-enhanced steam methane reforming
SMR: steam methane reforming
SMR-CaL-DRM: steam methane reforming coupled with calcium looping and dry reforming of methane
WGS: water gas shift
