# Supplementary material for: Integrated CO2 Capture and Utilization by Combining Calcium Looping with CH4 Reforming Processes: A Thermodynamic and Exergetic Approach
Source: Energy Fuels. 2024 Jun 21;38(13):11966–79. doi: 10.1021/acs.energyfuels.4c01462 (PMC11232036; doi:10.1021/acs.energyfuels.4c01462)
Supplement: Supplementary file 1 — ef4c01462_si_001.pdf [file ef4c01462_si_001.pdf]

Supplementary information

Integrated CO_2 capture and utilization by combining calcium looping with CH_4 reforming processes: A thermodynamic and exergetic approach

Theodoros Papalas[1,2], Andy N. Antzaras[1,], Angeliki A. Lemonidou[1,3]*

[1]Department of Chemical Engineering, Aristotle University of Thessaloniki, University Campus, 54124 Thessaloniki, Greece

[2]Department of Chemical Engineering and Biotechnology, University of Cambridge, Philippa Fawcett Drive, Cambridge CB3 0AS, United Kingdom

[3]Chemical process & Energy Resource Institute, CPERI/CERTH, 57001 Thermi, Thessaloniki, Greece

*Corresponding author

Andy N. Antzaras

Land line: +30 2310 996199

Fax: +30 2310 996184

Email: aantzara@cheng.auth.gr

Nomenclature

$A(K_i)$	pre-exponential factor of adsorption constant K_i for component i
$A(k_n)$	pre-exponential factor of Arrhenius parameter k_n for reaction n
$C_{CO2,eq}$	equilibrium concentration of CO_2
C_i	available molecules of component i per reaction volume
d_i	crystallite size of component i
E_n	activation energy of reaction n
K'	apparent constant of $CaCO_3$ decomposition model
$K_{eq,n}$	equilibrium constant of reaction n
K_i	adsorption constant of component i
k_n	Arrhenius parameter of reaction n
M_i	molecular weight of component i
$\dot{n}_{i,in}$	inlet molar flow of component i
$\dot{n}_{i,out}$	outlet molar flow of component i
P	pressure
P_i	partial pressure of component i
R	ideal gas constant
r_n	rate of reaction n expressed with reaction volume in denominator
r_n'	rate of reaction n expressed with catalyst weight in denominator
S_{BET}^{i}	BET surface area per mass of component i
S_{BET}^{tot}	BET surface area per mass of material
T	temperature
$V_{m,CaO}$	molar volume of CaO
w_i	weight fraction of component i
ΔH_i	enthalpy change of adsorption constant K_i for component i
ε	void fraction of reactor
ρ_{bed}	bed density
$\rho_{s,i}$	skeletal density of component i

S.1. Kinetic rate equations used for main simulations

Kinetic models of literature are used to describe the reactions that take place in the reformer and the calciner. Regarding the reformer, the *Langmuir – Hinshelwood* model of *Xu* and *Froment*[1] are employed for the kinetic rates of SMR (Eq. (S1)) and WGS (Eq. (S2)) reactions. The K_i terms present in the denominator of reaction rates refer to the adsorption constants of different components and can be expressed with a *Van't Hoff* equation, while **Table S1** lists the pre-exponential factors and enthalpy changes for all adsorption constants. The kinetic expressions used also incorporate the equilibrium constants for the SMR ($K_{eq,1}$) and WGS ($K_{eq,2}$) reactions, which can be found from Eq. (S3) and Eq. (S4) respectively.

$$r_1' = k_1 \times \frac{\dfrac{P_{CH_4} \times P_{H_2O}}{P_{H_2}^{2.5}} - \dfrac{P_{H_2}^{0.5} \times P_{CO}}{K_{eq,1}}}{\left(1 + K_{CO} \times P_{CO} + K_{H_2} \times P_{H_2} + K_{CH_4} \times P_{CH_4} + \dfrac{K_{H_2O} \times P_{H_2O}}{P_{H_2}}\right)^2} \tag{S1}$$

$$r_2' = k_2 \times \frac{\dfrac{P_{CO} \times P_{H_2O}}{P_{H_2}} - \dfrac{P_{CO_2}}{K_{eq,2}}}{\left(1 + K_{CO} \times P_{CO} + K_{H_2} \times P_{H_2} + K_{CH_4} \times P_{CH_4} + \dfrac{K_{H_2O} \times P_{H_2O}}{P_{H_2}}\right)^2} \tag{S2}$$

$$K_{eq,1}\,(bar^2) = \frac{P_{CO} \times P_{H_2}^3}{P_{CH_4} \times P_{H_2O}} = 6.14 \times 10^{13} \times e^{-28,116/T(K)} \tag{S3}$$

$$K_{eq,2}\,(-) = \frac{P_{CO_2} \times P_{H_2}}{P_{CO} \times P_{H_2O}} = 0.013 \times e^{4,577.8/T(K)} \tag{S4}$$

For the reactions described above, the reaction rate is expressed with catalyst weight in the denominator (r_n'). Each reaction rate r_n' is multiplied with the bed density ρ_{bed}, for the reaction rate to have the reaction volume in the denominator instead (r_n). In Aspen Plus, the reaction volume refers to the volume occupied by gas components inside the reactor. This is accounted by dividing with the void fraction ε (Eq. (S5)).

$$r_n = \frac{\rho_{bed} \times r_n'}{\varepsilon} \tag{S5}$$

Table S1. *Adsorption constants of gas components for SMR and WGS reactions*[1]

Van't Hoff equation: $K_i = A(K_i) \times e^{-\Delta H_i / R \times T}$			
Component i	$A(K_i)$	ΔH_i (kJ/mol)	Units of measurement
CH_4	6.65×10^{-4}	-38.3	$1/bar$
H_2O	1.77×10^{5}	+88.7	-
H_2	6.12×10^{-9}	-82.9	$1/bar$
CO	8.23×10^{-5}	-70.9	$1/bar$

The kinetic expressions of *Scaltsoyiannes et al.*[2,3] is used to predict the extent of the carbonation and calcination reactions. Eqs. (S6) and (S7) present the kinetic expressions for these reactions, which have undergone minor modification to consider the fraction of CaO or $CaCO_3$ in the reactors. Carbonation is known to proceed *via* a fast reaction of CO_2 with CaO on the surface, followed by the shift of the kinetically controlled stage of the reaction to the slower CO_2 diffusion through the formed carbonates.[4] The modified random pore model of *Scaltsoyiannes et al.* can predict the CaO conversion during the fast kinetically controlled regime,[2] while our sorption enhanced reforming experiments have shown that $CaZrO_3$-promoted CaO can attain 90% conversion in this regime.[5]

$$r_3 = \frac{k_3 \times M_{CaO} \times S_{BET}^{CaO}}{V_{m,CaO}} \times C_{CaO} \times (C_{CO_2} - C_{CO_2,eq}) \tag{S6}$$

$$r_4 = k_4 \times S_{BET}^{CaCO_3} \times M_{CaCO3} \times C_{CaCO3} \times \frac{C_{CO_2,eq} - C_{CO_2}}{(C_{CO_2,eq} + K_{eq,3} \times K' \times C_{CO_2})} \tag{S7}$$

Eq. (S7) contains a K' term, which can be expressed as a function of temperature (Eq. (S8)). Moreover, both carbonation and calcination models contain an equilibrium CO_2 concentration ($C_{CO_2,eq}$) term. The latter is related to the equilibrium constant ($K_{eq,4}$) of the reversible reaction and can be found from Eq. (S9), based on thermochemical data[6]

for $K_{eq,4}$ and the ideal gas law. Moreover, S_{BET}^{CaO} and $S_{BET}^{CaCO_3}$ of Eqs. (S6) and (S7) refer to the surface area of the carbonated and calcined material per mass of CaO and $CaCO_3$ respectively. For their retrieval, X-ray diffraction and N_2 adsorption are conducted to find the crystallite size of each phase i (d_i) and the total surface area (S_{BET}^{tot}). Scanning electron microscopy is also employed, which reveals an homogeneous dispersion of crystallites of all phases within a grain of a material. The results of all characterization methods can be found from our previous work.[7] Based on the obtained results and following the methodology of *Scaltsoyiannes et al.*,[2] the contribution of the surface area of each phase i (S_{BET}^{i}) to the total surface area is dependent on its weight fraction w_i (Eq. (S10)). By assuming spherical crystallites, the crystallite site of each phase is related to its skeletal density ($\rho_{s,i}$) and surface area according to Eq. (S11), while combining Eqs. (S10) and (S11) allows the retrieval of S_{BET}^{CaO} and $S_{BET}^{CaCO_3}$ terms (Eq. (S12)).

$$K'(1/bar) = 1.38 \times 10^{-3} \times e^{101/R \times T(K)} \tag{S8}$$

$$C_{CO_2,eq}(kmol/m^3) = \frac{K_{carb,eq}}{R \times T(K)} = \frac{4.975 \times 10^8}{T(K)} \times \exp\left(-\frac{20{,}474}{T(K)}\right) \tag{S9}$$

$$S_{BET}^{tot} = S_{BET}^{i} \times w_i + S_{BET}^{CaZrO_3} \times w_{CaZrO_3} + S_{BET}^{Ni} \times w_{Ni},\ i = \text{CaO or } CaCO_3 \tag{S10}$$

$$d_i = \frac{6}{\rho_{s,i} \times S_{BET}^{i}} \tag{S11}$$

$$S_{BET}^{i} = \frac{S_{BET}^{tot}}{w_i + \frac{d_{CaZrO3} \times \rho_{s,CaZrO_3}}{d_{CaO} \times \rho_{s,CaO}} + \frac{d_{Ni} \times \rho_{s,Ni}}{d_{CaO} \times \rho_{s,CaO}}}, i = CaO\ or\ CaCO_3 \tag{S12}$$

DRM and RWGS reactions occur simultaneously with calcination in the reactor of *Case 1*, whose extent is predicted by incorporating the kinetic equations (Eqs. (S13) and (S14)) of *Richardson* and *Paripatyadar*[8] in the simulation. Similarly to the *SMR* and *WGS* reactions, the rates are expressed with mass of catalyst in their denominator

and require modification according to Eq. (S5). The $K_{eq,5}$ and $K_{eq,6}$ terms refer to the equilibrium constants of the reactions, which can be retrieved from Eqs. (S15) and (S16) respectively. The K_i terms refer to the adsorption coefficients, with **Table S2** providing the pre-exponential factors and enthalpy changes needed for their calculation.

$$r_5' = k_5 \times K_{CH_4} \times K_{CO_{2,1}} \times \frac{P_{CH_4} \times P_{CO_2} - \frac{P_{H_2}^2 \times P_{CO}^2}{K_{eq,5}}}{\left(1 + K_{CH_4} \times P_{CH_4} + K_{CO_{2,1}} \times P_{CO_2}\right)^2} \tag{S13}$$

$$r_6' = k_6 \times K_{H_2} \times K_{CO_{2,2}} \times \frac{P_{H_2} \times P_{CO_2} - \frac{P_{CO} \times P_{H_2O}}{K_{eq,6}}}{\left(1 + K_{H_2} \times P_{H_2} + K_{CO_{2,2}} \times P_{CO_2}\right)^2} \tag{S14}$$

$$K_{eq,5}\,(bar^2) = \frac{P_{CO}^2 \times P_{H_2}^2}{P_{CH_4} \times P_{CO_2}} = 4.72 \times 10^{15} \times e^{-32{,}693/T(K)} \tag{S15}$$

$$K_{eq,6}\,(-) = \frac{P_{CO} \times P_{H_2O}}{P_{CO_2} \times P_{H_2}} = \left(K_{eq,2}\right)^{-1} = 76.923 \times e^{-4{,}577.8/T(K)} \tag{S16}$$

Table S2. *Adsorption constants of gas components for DRM and RWGS reactions*[8]

Van't Hoff equation: $K_i = A(K_i) \times e^{-\Delta H_j/R\times T}$			
Component i	$A(K_i)$	ΔH_i (kJ/mol)	Units of measurement
CH_4	2.57×10^{-2}	-40.684	$1/bar$
$CO_{2,1}$	2.58×10^{-2}	-37.641	$1/bar$
H_2	1.474	-9.262	$1/bar$
$CO_{2,2}$	5.695×10^{-1}	-6.025	$1/bar$

Finally for the SE-SMR process with the oxy-fuel calciner (*Case 3),* a power law kinetic model (Eq. (S17)) is used to describe the combustion of CH_4 with O_2.[9] **Table S3** provides the pre-exponential constants and activation energies for all reactions.

$$r_7 = k_7 \times T^{-1} \times C_{CH_4} \times C_{O_2} \tag{S17}$$

Table S3. *Arrhenius pre-exponential factors and activation energies*

Arrhenius equation: $k_n = A(k_i) \times e^{-E_n/_{R\times T}}$				
reaction n	$A(k_n)$	E_n (kJ/mol)	Units of measurement	Reference
1	1.17×10^{12}	240.1	$kmol/(kg \times s \times bar^{0.404})$	[1]
2	543.06	67.13	$kmol/(kg \times s \times bar)$	[1]
3	6.08×10^{4}	22.1	$m^4/(kmol \times s)$	[2]
4	18,860	210	$kmol/(m^2 \times s)$	[3]
5	1,290	102.065	$kmol/(kg \times s)$	[8]
6	350	81.030	$kmol/(kg \times s)$	[8]
7	3.552×10^{14}	130.5	$K \times m^3/(kmol \times s)$	[9]

S.2. Further results of thermodynamic analysis

For the results of the simulations presented in the main text, carbon formation has been neglected, while the thermodynamics of conventional DRM suggest that carbon formation should also be considered especially, for operation at low temperatures.[10,11] **Figure S1** illustrates the equilibrium outlet composition as a function of temperature, where calculations are performed by either neglecting or considering carbon as component in the simulation. When not accounting carbon formation (Figure S1a), CO and H_2 flows present a sigmoidal increase, accompanied with a gradual drop of CH_4 with increasing temperature. Above 700°C, the effect of temperature on the production and consumption rates of different components is milder and dictated only by the equilibrium of DRM and RWGS. Including carbon in the simulation (Figure S1b) reveals its gradual formation for temperatures up to ~660-680°C, accompanied with a higher and lower molar flow of H_2 and CH_4 respectively compared to the simulation without carbon, indicating a higher CH_4 conversion as a result of decomposition (Eq. (5)). The CO outlet flow is also slightly lower, as a combined result of reduced selectivity of CH_4 to DRM

and the CO disproportionation reaction (Eq. (6)). All outlet flows continue two different regimes, with the transition point, which corresponds to the end of $CaCO_3$ calcination, been shifted at slightly higher temperatures (720°C), since CH_4 selectivity toward carbon formation reduces the calcination driving force. The negative effect of carbon formation stresses the need to use carbon-resistant catalysts for practical application of the process.

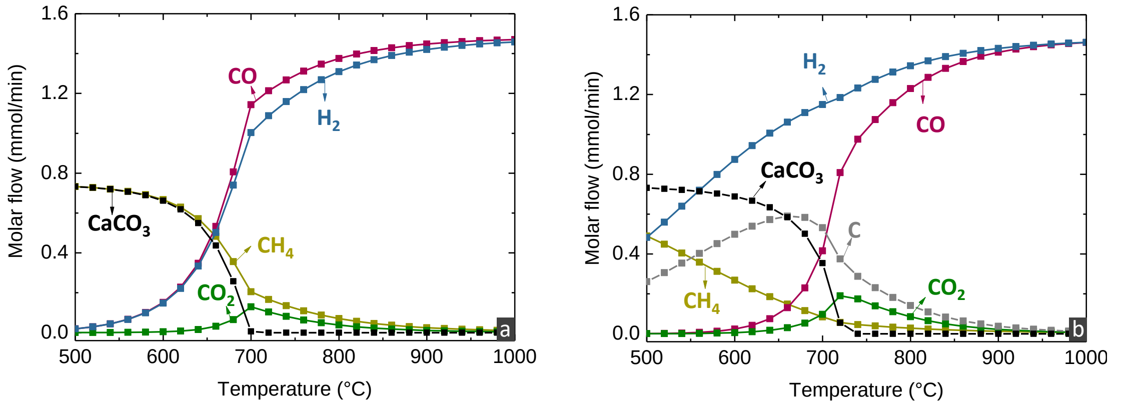

Figure S1. *Molar flow composition (dry conditions) of the outlet of the calcination/DRM stage as a function of temperature for thermodynamic calculations (a) without and (b) with solid carbon formation (P=1 bar, $CH_4/CaCO_3$=1)*

Figure S2 displays the effect of different operating parameters on the performance indicators while accounting carbon formation. Temperature comprises an important parameter, with the gradually increasing formation of carbon up to ~660-680°C being accompanied with an enhanced CH_4 conversion, purity and H_2/CO molar ratio of syngas. Complete $CaCO_3$ conversion is reached at ~720°C, a slightly higher temperature than the simulations without carbon formation (Figure 2), proving that carbon has a negative effect on the calcination driving force. This is highlighted by the lower *in situ* utilization of CO_2 at the temperature of complete $CaCO_3$ calcination compared to the simulations without carbon formation (~70% instead of ~80%). Regarding the effect of pressure, similar degrees of $CaCO_3$ calcination and *in situ* CO_2 utilization are attained compared to the simulations with no carbon deposition (Figure 3). The conversion of

CH_4 decreases with pressure, while being retained at high values due to its consumption toward carbon formation, along with the purity and H_2/CO molar ratio of syngas. Finally, regarding the $CH_4/CaCO_3$ molar ratio, since less CH_4 is available for DRM and the enhancement of calcination is limited, higher $CH_4/CaCO_3$ molar ratios are required to decompose $CaCO_3$.

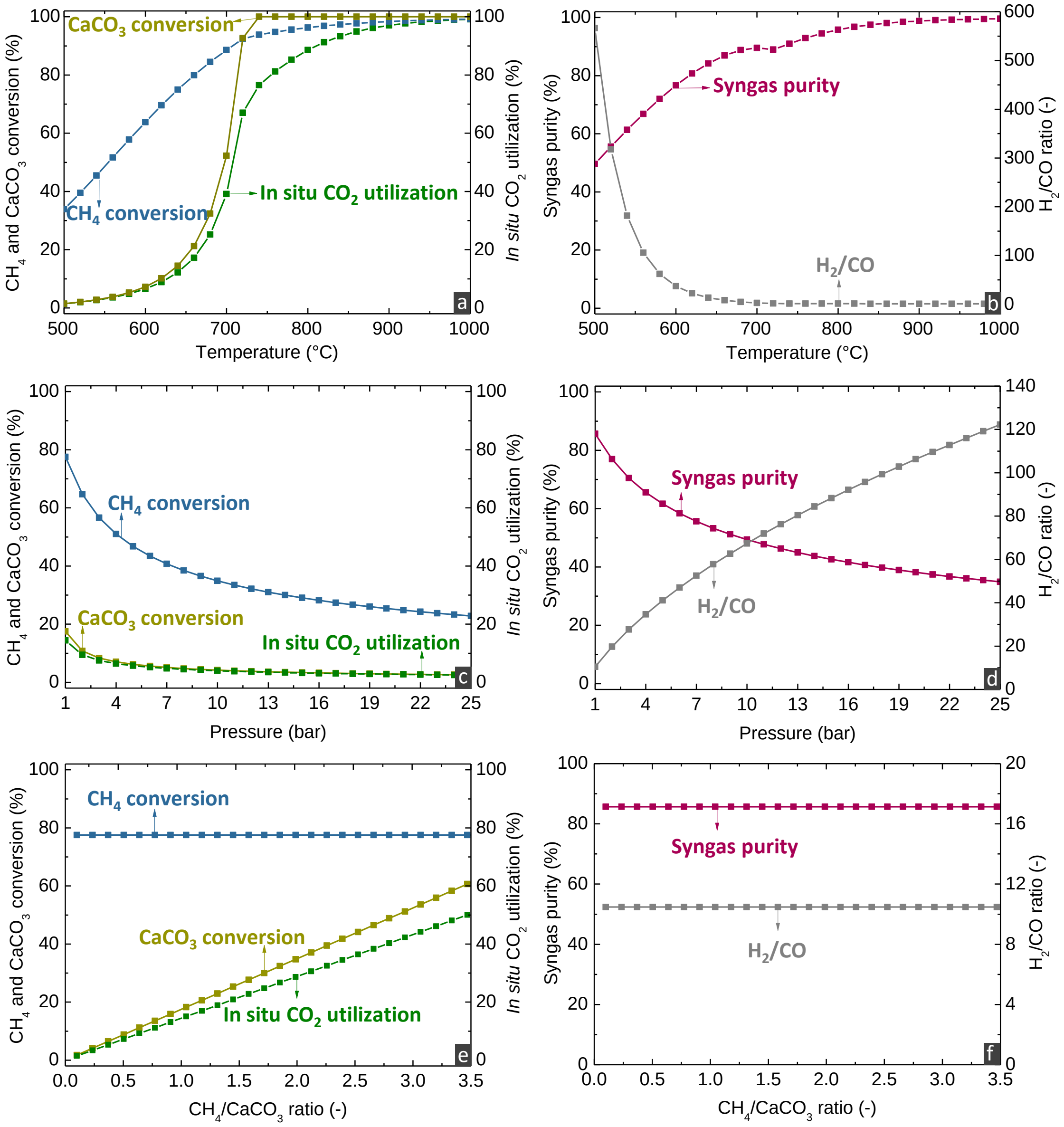

Figure S2. *Effect of (a), (b) temperature, (c), (d) pressure and (e), (f) $CH_4/CaCO_3$ molar ratio on $CaCO_3$ conversion, CH_4 conversion, in situ CO_2 utilization, purity and H_2/CO molar ratio of produced syngas in the calcination/DRM stage when accounting solid carbon formation (<u>standard conditions</u>: T=650°C, P=1 bar, $CH_4/CaCO_3$=1)*

Performance indicators displayed in Figures 2, 3, 4 and S2 were calculated using the outlet molar flow composition of the RGIBBS reactor of the Aspen Plus software. Figure S1 displayed the molar flow composition with varying temperature, while **Figures S3** and **S4** demonstrate the composition as a function of pressure and $CH_4/CaCO_3$ ratio. These figures are provided for verification purposes, since their discussion would be very similar to the respective ones with the performance indicators. For all simulations, the solid inlet stream of the *RGIBBS* model consists of 26mol% CaO and 74mol% $CaCO_3$. This $CaO/CaCO_3$ ratio corresponds to the outlet of a sorption enhanced steam methane reformer operating at the optimum conditions (650°C, 1 bar, H_2O to CH_4 molar ratio of 3 and CaO to CH_4 molar ratio of 1), as defined previously.[12]

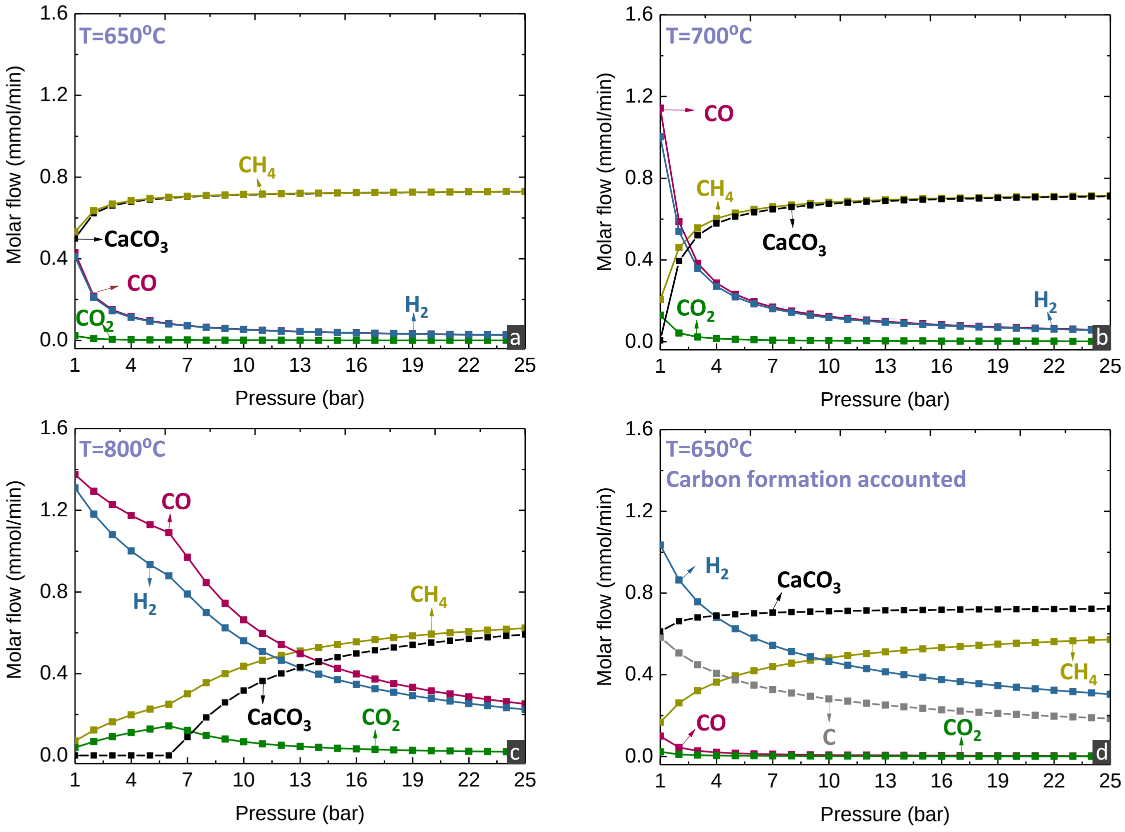

Figure S3. *Molar flow composition (dry conditions) of the outlet of the calcination/DRM stage as a function of pressure for thermodynamic calculations without solid carbon formation at (a) 650, (b) 700 and (c) 800°C and (d) with solid carbon formation at 650°C ($CH_4/CaCO_3$=1)*

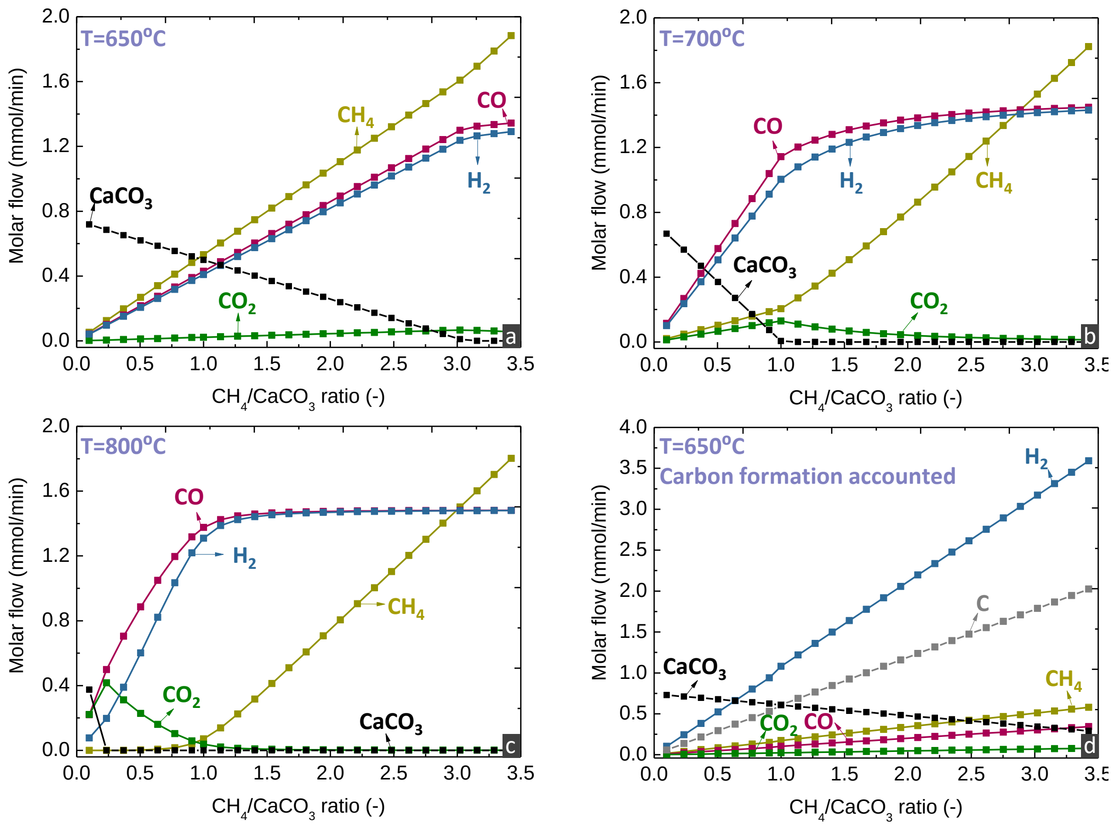

Figure S4. *Molar flow composition (dry conditions) of the outlet of the calcination/DRM stage as a function of $CH_4/CaCO_3$ molar ratio for thermodynamic calculations without solid carbon formation at (a) 650, (b) 700 and (c) 800°C and (d) with solid carbon formation at 650°C (P=1 bar)*

S.3. Further data of conceptual process design

Figure 5 presents the performance of the reformer in terms of attained CH_4 conversion, H_2 purity and CO_2 capture, which have been defined in our previous work on the performance of the reforming reactor.[12] CH_4 conversion is expressed (Eq. (S18)) as the difference between the inlet and outlet molar flows of CH_4, divided by the inlet molar flow of CH_4. CaO conversion is defined similarly (Eq. (S19)) as the difference between the inlet and outlet molar flows of CaO, divided by the inlet molar flow of CaO. Finally, H_2 purity is defined (Eq. (S20)) in dry basis as the outlet molar flow of H_2, divided by the total molar flow in the outlet stream.

$$CH_4\ conversion\ (\%) = 100 \times \frac{\dot{n}_{CH_{4,in}} - \dot{n}_{CH_{4,out}}}{\dot{n}_{CH_{4,in}}} \tag{S18}$$

$$CaO\ conversion\ (\%) = 100 \times \frac{\dot{n}_{CaO_{in}} - \dot{n}_{CaO_{out}}}{\dot{n}_{CaO_{in}}} \tag{S19}$$

$$H_2\ purity\ (\%) = 100 \times \frac{\dot{n}_{H_{2,out}}}{\dot{n}_{H_{2,out}} + \dot{n}_{CO_{,out}} + \dot{n}_{CH_{4,out}} + \dot{n}_{CO_{2,out}}} \tag{S20}$$

Tables S4, **S5** and **S6** display the composition and operating conditions for the different streams of the flow diagrams for *Cases 1*, *2* and *3*. The tabulated data is used to calculate the different performance indicators presented in Figure 5 and the exergy balances of each equipment module and of the whole process (Figure 6 and Table 4).

Table S4. *Molar flows, composition, temperature and pressure of each stream of the flow diagram for the integrated SMR-CaL-DRM process (Case 1)*

**Stream**		**101**	**102**	**103**	**104**	**105**	**106**	**107**	**108**	**109**	**201**	**202**	**203**	**204**	**205**	**206**	**207**	**208**	**209**
**Gases (kmol/hr)**		**147.4**	**287.2**	**442.2**	**442.2**	**442.2**	**268.4**	**367.3**	**332.2**	**332.2**	**589.6**	**589.6**	**589.6**	**733.5**	**733.5**	**128.1**	**128.1**	**128.1**	**128.1**
**mol. %**	**CH_4**	100.0	–	–	–	–	–	2.5	–	–	25.0	25.0	25.0	1.5	1.5	100.0	100.0	100.0	100.0
	**H_2O**	–	100.0	100.0	100.0	100.0	–	4.5	30.6	30.6	75.0	75.0	75.0	23.8	23.8	–	–	–	–
	**CO**	–	–	–	–	–	–	1.0	–	–	–	–	–	0.5	0.5	–	–	–	–
	**H_2**	–	–	–	–	–	–	18.1	–	–	–	–	–	73.6	73.6	–	–	–	–
	**CO_2**	–	–	–	–	–	–	0.8	4.8	4.8	–	–	–	0.5	0.5	–	–	–	–
	**O_2**	–	–	–	–	–	21.0	15.4	0.8	0.8	–	–	–	–	–	–	–	–	–
	**N_2**	–	–	–	–	–	79.0	57.7	63.8	63.8	–	–	–	–	–	–	–	–	–
**T (°C)**		15	15	22	102	170	20	11	1000	122	129	470	630	601	259	15	239	515	573
**P (bar)**		1.0	1.0	1.0	1.0	1.0	1.0	1.0	1.0	1.0	1.0	1.0	0.9	1.0	0.9	1.0	1.0	1.0	1.0

**Stream**		**210**	**211**	**212**	**301**	**302**	**303**	**304**	**305**	**306**	**307**	**308**	**309**	**310**	**311**	**312**	**313**	**314**	**315**
**Gases (kmol/hr)**		**128.1**	**505.6**	**505.6**	**733.5**	**733.5**	**154.5**	**579.0**	**579.0**	**458.7**	**98.9**	**10.2**	**26.3**	**36.5**	**32.9**	**32.9**	**505.6**	**505.6**	**505.6**
**mol. %**	**CH_4**	100.0	0.7	0.7	1.5	1.5	–	1.9	1.9	–	9.4	9.4	–	2.6	–	–	0.7	0.7	0.7
	**H_2O**	–	0.3	0.3	23.8	23.8	100.0	3.5	3.5	–	16.7	16.7	–	4.7	31.8	31.8	0.3	0.3	0.3
	**CO**	–	49.7	49.7	73.6	73.6	–	0.8	0.8	–	3.7	3.7	–	1.0	–	–	49.7	49.7	49.7
	**H_2**	–	49.0	49.0	0.5	0.5	–	93.2	93.2	100.0	67.3	67.3	–	18.8	–	–	49.0	49.0	49.0
	**CO_2**	–	0.3	0.3	0.5	0.5	–	0.6	0.6	–	2.9	2.9	–	0.8	5.0	5.0	0.3	0.3	0.3
	**O_2**	–	–	–	–	–	–	–	–	–	–	–	21.0	15.1	–	–	–	–	–
	**N_2**	–	–	–	–	–	–	–	–	–	–	–	79.0	57.0	63.2	63.2	–	–	–
**T (°C)**		800	800	533	207	30	30	30	30	30	20	20	20	17	1000	593	408	147	30
**P (bar)**		1.0	1.0	1.0	0.8	0.9	1.0	1.0	25.0	25.0	1.3	1.3	1.0	1.0	1.0	1.0	0.9	0.9	0.9

Utilities

**Stream**		**Flare**	**U-301**	**U-302**	**U-303**	**U-304**	**U-305**	**U-306**	**U-307**	**U-308**	**U-309**
**Gases (kmol/hr)**		**11.2**	**5889.0**	**5889.0**	**91.0**	**91.0**	**91.0**	**2683.0**	**2683.0**	**1061.3**	**1061.3**
**mol. %**	**CH_4**	9.4	–	–	–	–	–	–	–	–	–
	**H_2O**	16.7	100.0	100.0	100.0	100.0	100.0	100.0	100.0	100.0	100.0
	**CO**	3.7	–	–	–	–	–	–	–	–	–
	**H_2**	67.3	–	–	–	–	–	–	–	–	–
	**CO_2**	2.9	–	–	–	–	–	–	–	–	–
	**O_2**	–	–	–	–	–	–	–	–	–	–
	**N_2**	–	–	–	–	–	–	–	–	–	–
**T (°C)**		20	20	40	125	250	129	20	40	20	40
**P (bar)**		1.3	1.0	0.9	2.5	39.7	2.5	1.0	0.9	1.0	0.9

**Stream**		**213**	**214**
**Solids (kmol/hr)**		**188.8**	**188.8**
**mol. %**	**CaO**	78.0	10.2
	**$CaCO_3$**	–	67.8
	**Ni**	9.8	9.8
	**$CaZrO_3$**	12.2	12.2
**T (°C)**		900	601
**P (bar)**		1.0	1.0

Table S5. *Molar flows, composition, temperature and pressure of each stream of the flow diagram for the SE-SMR process with solar calciner (Case 2)*

Stream		101	102	103	104	105	106	107	108	109	201	202	203	204	205	206	207	208	209
Gases (kmol/hr)		147.4	287.2	442.2	442.2	442.2	268.6	367.2	332.2	332.3	589.6	589.6	589.6	733.3	733.3	128.1	128.1	128.1	128.1
mol. %	CH_4	100.0	–	–	–	–	–	2.5	–	–	25.0	25.0	25.0	1.5	1.5	–	–	–	–
	H_2O	–	100.0	100.0	100.0	100.0	–	4.5	30.6	30.6	75.0	75.0	75.0	23.8	23.8	–	–	–	–
	CO	–	–	–	–	–	–	1.0	–	–	–	–	–	0.06	0.06	–	–	–	–
	H_2	–	–	–	–	–	–	18.1	–	–	–	–	–	73.5	73.5	–	–	–	–
	CO_2	–	–	–	–	–	–	0.8	4.7	4.7	–	–	–	0.5	0.5	100.0	100.0	100.0	100.0
	O_2	–	–	–	–	–	21.0	15.4	0.8	0.8	–	–	–	–	–	–	–	–	–
	N_2	–	–	–	–	–	79.0	57.7	63.9	63.9	–	–	–	–	–	–	–	–	–
T (°C)		15	15	22	102	170	20	11	1000	122	129	470	585	600	258	15	238	678	703
P (bar)		1.0	1.0	1.0	1.0	1.0	1.0	1.0	1.0	1.0	1.0	1.0	0.9	1.0	0.9	1.0	1.0	1.0	1.0

Stream		210	211	212	301	302	303	304	305	306	307	308	309	310	311	312	313	314	315
Gases (kmol/hr)		128.1	256.2	256.2	733.3	733.3	154.8	578.5	578.5	458.3	98.6	5.8	15.2	21.0	18.9	18.9	256.2	256.2	256.2
mol. %	CH_4	–	–	–	1.5	1.5	–	1.9	1.9	–	9.5	9.5	–	2.6	–	–	-	-	-
	H_2O	–	–	–	23.8	23.8	100.0	3.5	3.5	–	16.7	16.7	–	4.7	31.8	31.8	-	-	-
	CO	–	–	–	0.06	0.06	–	0.8	0.8	–	3.6	3.6	–	1.0	–	–	-	-	-
	H_2	–	–	–	73.5	73.5	–	93.2	93.2	100.0	67.3	67.3	–	18.7	–	–	-	-	-
	CO_2	100.0	100.0	100.0	0.5	0.5	–	0.6	0.6	–	2.9	2.9	–	0.8	4.9	4.9	100.0	100.0	100.0
	O_2	–	–	–	–	–	–	–	–	–	–	–	21.0	15.2	–	–	–	–	–
	N_2	–	–	–	–	–	–	–	–	–	–	–	79.0	57.0	63.3	63.3	–	–	–
T (°C)		900	900	698	198	30	30	30	30	30	20	20	20	11	1000	674	474	145	30
P (bar)		1.0	1.0	1.0	0.8	0.9	1.0	1.0	25.0	25.0	1.3	1.3	1.0	1.0	1.0	1.0	0.9	0.9	0.9

Utilities

Stream		Flare	U-301	U-302	U-303	U-304	U-305	U-306	U-307	U-308	U-309
Gases (kmol/hr)		15.8	5772.0	5772.0	97.0	97.0	97.0	2860.0	2860.0	729.5	729.5
mol. %	CH_4	9.5	–	–	–	–	–	–	–		
	H_2O	16.7	100.0	100.0	100.0	100.0	100.0	100.0	100.0	100.0	100.0
	CO	3.6	–	–	–	–	–	–	–	–	–
	H_2	67.3	–	–	–	–	–	–	–	–	–
	CO_2	2.9	–	–	–	–	–	–	–	–	–
	O_2	–	–	–	–	–	–	–	–	–	–
	N_2	–	–	–	–	–	–	–	–	–	–
T (°C)		20	20	40	125	250	129	20	40	20	40
P (bar)		1.3	1.0	0.9	2.5	39.7	2.5	1.0	0.9	1.0	0.9

Stream		213	214
Solids (kmol/hr)		188.8	188.8
mol. %	CaO	78	10.2
	$CaCO_3$	–	67.8
	Ni	9.8	9.8
	$CaZrO_3$	12.2	12.2
T (°C)		900	600
P (bar)		1.0	1.0

Table S6. *Molar flows, composition, temperature and pressure of each stream of the flow diagram for the SE-SMR process with oxy-fuel calciner (Case 3)*

**Stream**		**101**	**102**	**103**	**104**	**105**	**106**	**107**	**108**	**109**	**201**	**202**	**203**	**204**	**205**	**206**	**207**	**208**	**209**
**Gases (kmol/hr)**		**147.4**	**287.2**	**442.2**	**442.2**	**442.2**	**268.1**	**366.7**	**331.8**	**331.8**	**589.6**	**589.6**	**589.6**	**733.3**	**733.3**	**107.1**	**107.1**	**107.1**	**107.1**
**mol. %**	**CH_4**	100.0	–	–	–	–	–	2.5	–	–	25.0	25.0	25.0	1.5	1.5	**33.3**	33.6	33.6	33.6
	**H_2O**	–	100.0	100.0	100.0	100.0	–	4.5	30.6	30.6	75.0	75.0	75.0	23.8	23.8	–	–	–	–
	**CO**	–	–	–	–	–	–	1.0	–	–	–	–	–	0.06	0.06	–	–	–	–
	**H_2**	–	–	–	–	–	–	18.1	–	–	–	–	–	73.5	73.5	–	–	–	–
	**CO_2**	–	–	–	–	–	–	0.8	4.7	4.7	–	–	–	0.5	0.5	–	–	–	–
	**O_2**	–	–	–	–	–	21.0	15.4	0.8	0.8	–	–	–	–	–	66.7	66.7	66.7	66.7
	**N_2**	–	–	–	–	–	79.0	57.7	63.9	63.9	–	–	–	–	–	–	–	–	–
**T (°C)**		15	15	22	102	170	20	11	1000	122	129	470	585	600	258	15	238	625	654
**P (bar)**		1.0	1.0	1.0	1.0	1.0	1.0	1.0	1.0	1.0	1.0	1.0	0.9	1.0	0.9	1.0	1.0	1.0	1.0

**Stream**		**210**	**211**	**212**	**301**	**302**	**303**	**304**	**305**	**306**	**307**	**308**	**309**	**310**	**311**	**312**	**313**	**314**	**315**
**Gases (kmol/hr)**		**107.1**	**235.2**	**235.2**	**733.3**	**733.3**	**154.8**	**578.5**	**578.5**	**458.3**	**98.4**	**3.6**	**9.5**	**13.1**	**11.9**	**11.9**	**235.2**	**235.2**	**235.2**
**mol. %**	**CH_4**	33.6	-	-	1.5	1.5	–	1.9	1.9	–	9.5	9.5	–	2.6	–	–	-	-	-
	**H_2O**	–	30.4	30.4	23.8	23.8	100.0	3.5	3.5	–	16.7	16.7	–	4.7	31.8	31.8	30.4	30.4	30.4
	**CO**	–	-	-	0.06	0.06	–	0.8	0.8	–	3.6	3.6	–	1.0	–	–	-	-	-
	**H_2**	–	-	-	73.5	73.5	–	93.2	93.2	100.0	67.3	67.3	–	18.7	–	–	-	-	-
	**CO_2**	–	69.6	69.6	0.5	0.5	–	0.6	0.6	–	2.9	2.9	–	0.8	4.9	4.9	69.6	69.6	69.6
	**O_2**	66.7	–	–	–	–	–	–	–	–	–	–	21.0	15.2	–	–	–	–	–
	**N_2**	–	–	–	–	–	–	–	–	–	–	–	79.0	57.0	63.3	63.3	–	–	–
**T (°C)**		800	900	645	222	30	30	30	30	30	20	20	20	11	1000	674	493	223	30
**P (bar)**		1.0	1.0	1.0	0.8	0.9	1.0	1.0	25.0	25.0	1.3	1.3	1.0	1.0	1.0	1.0	0.9	0.9	0.9

Utilities

**Stream**		**Flare**	**U-301**	**U-302**	**U-303**	**U-304**	**U-305**	**U-306**	**U-307**	**U-308**	**U-309**
**Gases (kmol/hr)**		**18.2**	**6117.0**	**6117.0**	**82.0**	**82.0**	**82.0**	**2065.5**	**2065.5**	**7043.9**	**7043.9**
**mol. %**	**CH_4**	9.5	–	–	–	–	–	–	–	–	–
	**H_2O**	16.7	100.0	100.0	100.0	100.0	100.0	100.0	100.0	100.0	100.0
	**CO**	3.6	–	–	–	–	–	–	–	–	–
	**H_2**	67.3	–	–	–	–	–	–	–	–	–
	**CO_2**	2.9	–	–	–	–	–	–	–	–	–
	**O_2**	–	–	–	–	–	–	–	–	–	–
	**N_2**	–	–	–	–	–	–	–	–	–	–
**T (°C)**		20	20	40	125	250	129	20	40	20	40
**P (bar)**		1.3	1.0	0.9	2.5	39.7	2.5	1.0	0.9	1.0	0.9

**Stream**		**213**	**214**
**Solids (kmol/hr)**		**188.8**	**188.8**
**mol. %**	**CaO**	78	10.2
	**$CaCO_3$**	–	67.8
	**Ni**	9.8	9.8
	**$CaZrO_3$**	12.2	12.2
**T (°C)**		800	600
**P (bar)**		1.0	1.0

References

(1) Xu, J.; Froment, G. F. Methane Steam Reforming, Methanation and Water-gas Shift: I. Intrinsic Kinetics. *AIChE J.* **1989**, *35* (1), 88–96. https://doi.org/10.1002/aic.690350109.

(2) Scaltsoyiannes, A.; Antzaras, A.; Koilaridis, G.; Lemonidou, A. Towards a Generalized Carbonation Kinetic Model of CaO-Based Materials Using a Modified Random Pore Model. *Chem. Eng. J.* **2021**, *407*, 127207. https://doi.org/10.1016/j.cej.2020.127207.

(3) Scaltsoyiannes, A.; Lemonidou, A. $CaCO_3$ Decomposition for Calcium-Looping Applications: Kinetic Modeling in a Fixed-Bed Reactor. *Chem. Eng. Sci. X* **2020**, *8*, 100071. https://doi.org/10.1016/j.cesx.2020.100071.

(4) Alvarez, D.; Abanades, J. C. Determination of the Critical Product Layer Thickness in the Reaction of CaO with CO_2. *Ind. Eng. Chem. Res.* **2005**, *44* (15), 5608–5615. https://doi.org/10.1021/ie050305s.

(5) Antzara, A.; Heracleous, E.; Lemonidou, A. A. Energy Efficient Sorption Enhanced-Chemical Looping Methane Reforming Process for High-Purity H_2 Production: Experimental Proof-of-Concept. *Appl. Energy* **2016**, *180*, 457–471. https://doi.org/10.1016/j.apenergy.2016.08.005.

(6) *Thermochemical Data of Pure Substances*; Barin, I., Ed.; Wiley-VCH Verlag GmbH: Weinheim, Germany, 1995. https://doi.org/10.1002/9783527619825.

(7) Papalas, T.; Lypiridis, D.; Antzaras, A.; Lemonidou, A. A. Experimental Investigation of Integrated CO_2 Capture and Conversion to Syngas via Calcium Looping Coupled with Dry Reforming of CH_4. *Chem. Eng. J.* **2024**, *485*, 149866. https://doi.org/10.1016/j.cej.2024.149866.

(8) Richardson, J. T.; Paripatyadar, S. A. Carbon Dioxide Reforming of Methane

with Supported Rhodium. *Appl. Catal.* **1990**, *61* (1), 293–309. https://doi.org/10.1016/S0166-9834(00)82152-1.

(9) de Souza-Santos, M. L. *Solid Fuels Combustion and Gasification*, 2nd ed.; CRC Press: Boca Raton, 2010. https://doi.org/10.1201/9781420047509.

(10) Jensen, C.; Duyar, M. S. Thermodynamic Analysis of Dry Reforming of Methane for Valorization of Landfill Gas and Natural Gas. *Energy Technol.* **2021**, *9* (7), 1–12. https://doi.org/10.1002/ente.202100106.

(11) Nikoo, M. K.; Amin, N. A. S. Thermodynamic Analysis of Carbon Dioxide Reforming of Methane in View of Solid Carbon Formation. *Fuel Process. Technol.* **2011**, *92* (3), 678–691. https://doi.org/10.1016/j.fuproc.2010.11.027.

(12) Antzara, A.; Heracleous, E.; Bukur, D. B.; Lemonidou, A. A. Thermodynamic Analysis of Hydrogen Production via Chemical Looping Steam Methane Reforming Coupled with In Situ CO_2 Capture. *Int. J. Greenh. Gas Control* **2015**, *32*, 115–128. https://doi.org/10.1016/j.ijggc.2014.11.010.
